# Design Strategies for Aqueous Zinc Metal Batteries with High Zinc Utilization: From Metal Anodes to Anode-Free Structures

**DOI:** 10.1007/s40820-023-01304-1

**Published:** 2024-01-04

**Authors:** Xianfu Zhang, Long Zhang, Xinyuan Jia, Wen Song, Yongchang Liu

**Affiliations:** 1 School of Materials Science and Engineering, University of Science and Technology Beijing, 30 College Road, Beijing, 100083 People’s Republic of China; 2 Beijing Advanced Innovation Center for Materials Genome Engineering, Institute for Advanced Materials and Technology, State Key Laboratory for Advanced Metals and Materials, University of Science and Technology Beijing, Beijing, 100083 People’s Republic of China

**Keywords:** Aqueous zinc metal batteries, Zinc anodes, High zinc utilization, Depth of discharge, Anode-free structures

## Abstract

Aqueous zinc metal batteries (AZMBs) are promising candidates for next-generation energy storage due to the excellent safety, environmental friendliness, natural abundance, high theoretical specific capacity, and low redox potential of zinc (Zn) metal. However, several issues such as dendrite formation, hydrogen evolution, corrosion, and passivation of Zn metal anodes cause irreversible loss of the active materials. To solve these issues, researchers often use large amounts of excess Zn to ensure a continuous supply of active materials for Zn anodes. This leads to the ultralow utilization of Zn anodes and squanders the high energy density of AZMBs. Herein, the design strategies for AZMBs with high Zn utilization are discussed in depth, from utilizing thinner Zn foils to constructing anode-free structures with theoretical Zn utilization of 100%, which provides comprehensive guidelines for further research. Representative methods for calculating the depth of discharge of Zn anodes with different structures are first summarized. The reasonable modification strategies of Zn foil anodes, current collectors with pre-deposited Zn, and anode-free aqueous Zn metal batteries (AF-AZMBs) to improve Zn utilization are then detailed. In particular, the working mechanism of AF-AZMBs is systematically introduced. Finally, the challenges and perspectives for constructing high-utilization Zn anodes are presented.

## Highlights

- Representative methods for calculating the depth of discharge of different Zn anodes are introduced.
- Recent advances of aqueous Zn metal batteries with high Zn utilization are reviewed and categorized according to Zn anodes with different structures.
- The working mechanism of anode-free aqueous Zn metal batteries is introduced in detail, and different modification strategies for anode-free aqueous Zn metal batteries are summarized.

## Graphical abstract

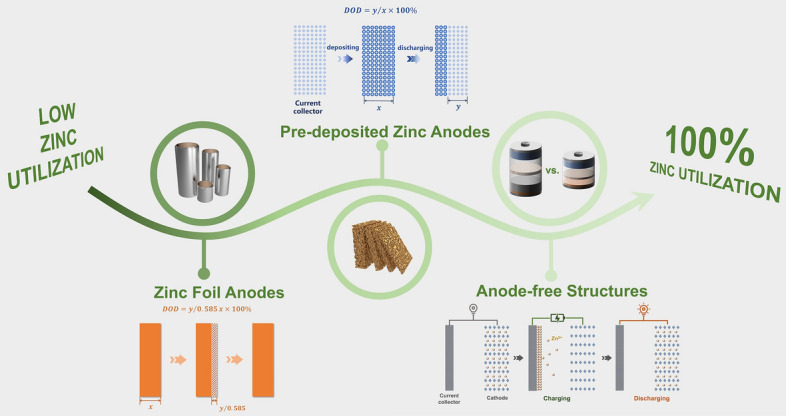

## Introduction

Lithium-ion batteries (LIBs) have shown remarkable success for use in portable electronic devices and electric vehicles owing to their high energy densities and long lifespans [1–4]. However, further application of LIBs is limited by concerns about their organic electrolytes, inadequate lithium reserves, and high costs [5–7]. Consequently, it is necessary to develop alternative secondary batteries to replace LIBs [8, 9]. Aqueous zinc metal batteries (AZMBs) have become competitive candidates due to the excellent theoretical capacities (820 mAh g^−1^ and 5855 mAh cm^−3^) and low electrochemical potentials (− 0.76 V vs. standard hydrogen electrode) of zinc (Zn) metal anodes, abundant Zn resources, and intrinsic security and high ionic conductivity of aqueous electrolytes (~ 1 S cm^−1^ vs. 1–10 mS cm^−1^ of organic electrolytes) [10–16]. However, serious issues of Zn metal anodes, such as hydrogen evolution reaction (HER), corrosion, passivation, and dendrite growth, lead to poor reversibility, unstable cycling life, and even short-circuited failure [17–23]. These issues significantly impede practical application of the AZMBs. Various stabilization strategies have been suggested for Zn metal anodes, including surface modification, structure optimization, electrolyte engineering, and separator design, to overcome the issues mentioned above [24–31]. Nevertheless, these studies have yet to achieve a high Zn utilization due to the use of far excess Zn [32]. To compensate for the irreversible loss of Zn and enhance the cycling stability of the charge/discharge process, researchers typically construct Zn metal anodes with excess Zn (thickness of Zn foil ≥ 100 μm) and low areal capacities (1–5 mAh cm^−2^), resulting in a high capacity ratio for the negative electrode to the positive electrode (N/P, > 50) and a low depth of discharge (DOD) (< 10%) [33].

The depth of discharge (DOD) is the percentage of the capacity involved in the electrode reaction relative to the overall capacity of the Zn metal anode:

1 $${\text{DOD}} = \frac{{C_{{{\text{Zn}},{\text{reactive}}}} }}{{C_{{{\text{Zn}},{\text{overall}}}} }} \times 100\%$$

The DOD is an important metric that reflects the Zn utilization and the serviceability of the Zn metal anode under practical conditions. Meanwhile, the DOD is an essential criterion for objectively evaluating the performance of AZMBs. Consequently, according to Eq. (1), reducing the amount of Zn used in the anode is an effective strategy to improve the Zn utilization.

In previous studies, excess Zn has been commonly present in the form of thick Zn foil (thickness ≥ 100 μm) [34–41]. The excess Zn continuously replenishes the active Zn to overcome losses due to “dead Zn” and byproducts, and this practice results in a deceptive cycling lifespan and impractical Coulombic efficiency (CE) [13, 42]. Additionally, the use of excess Zn raises the cost of the battery and reduces the actual energy density (calculated from the full cell mass) [43, 44]. When Zn is no longer an unlimited supplement, it is essential to inhibit the growth of Zn dendrites and reduce the formation of byproducts [45]. There have been several strategies for constructing Zn anodes with high Zn utilization. The most direct way to improve the Zn utilization is to control the active material within a reasonable range by reducing the thickness of the Zn foil or by using a pre-deposited Zn anode.

**Fig. 1:**
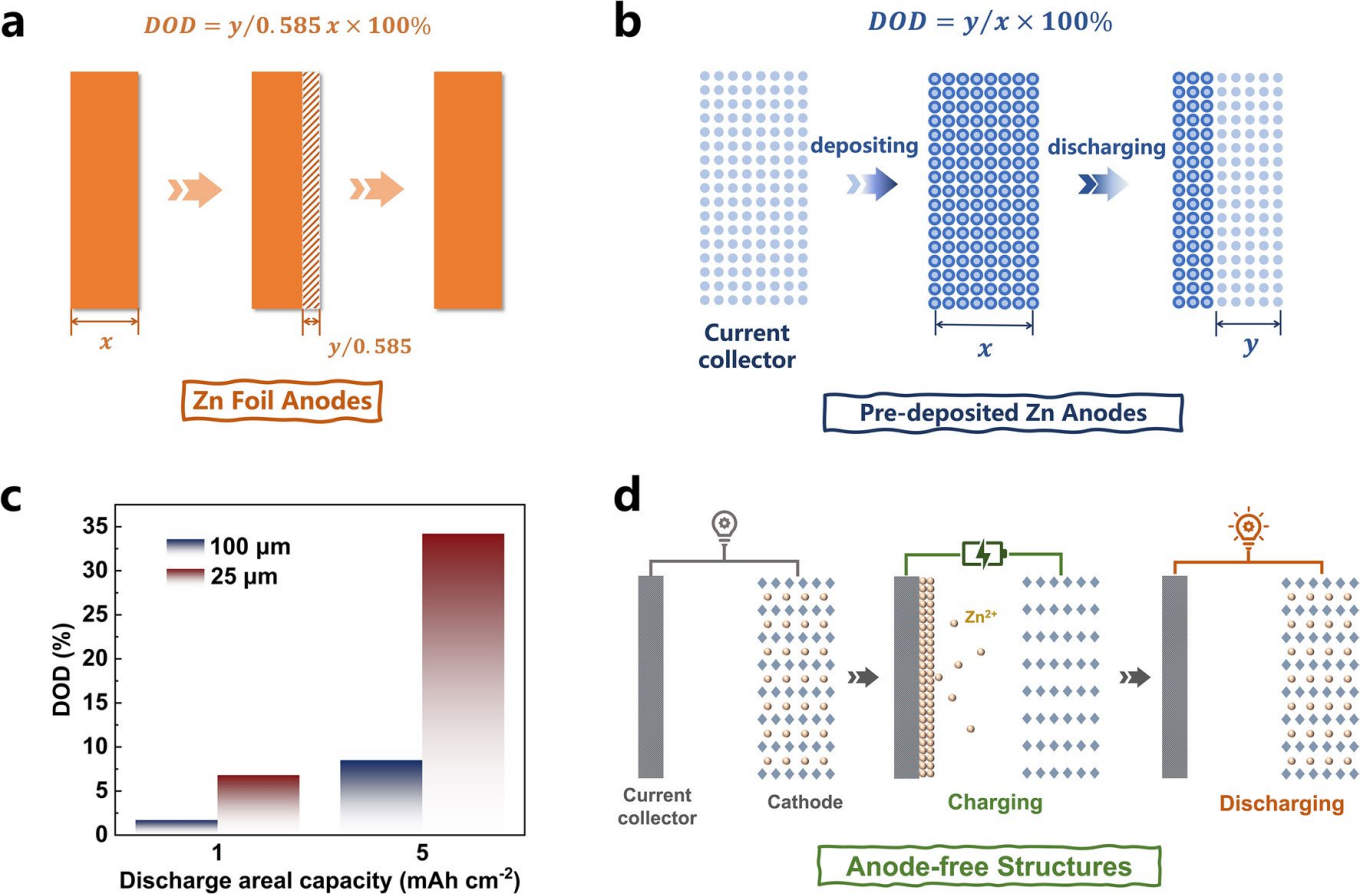
The schematic illustrations of the calculation of DOD for **a** Zn foil anode and **b** pre-deposited Zn anode. **c** DOD comparison of Zn foils with different thicknesses under the same areal discharge capacity. **d** Schematic diagram of the charging and discharging process of the assembled anode-free Zn metal battery

The formula used to calculate the DOD for a Zn metal anode using Zn foil is as follows:

2 $${\text{DOD}} = \frac{y}{{C_{{{\text{Zn}},{\text{volume}}}} \cdot x \times 10^{ - 4} }} \times 100\% = y/0.585x \times 100\%$$

where *x* (μm) is the thickness of the Zn foil and *y* (mAh cm^−2^) represents the Zn areal capacity used in electrochemical testing (Fig. 1a).

For Zn anodes using pre-deposited Zn,

3 $${\text{DOD}} = \frac{y}{{C_{{{\text{Zn}}, {\text{mass}}}} \cdot m \times 10^{ - 3} }} \times 100\% = y/x \times 100\%$$

where *x* (mAh cm^−2^) is the pre-deposited Zn capacity, *y* (mAh cm^−2^) is the Zn capacity used during electrochemical testing, and *m* (mg cm^−2^) is the pre-deposited Zn mass loading (Fig. 1b).

The theoretical mass capacity (*C*_Zn,mass_) and the theoretical volume capacity (*C*_Zn,volume_) are described in the equations below:

4 $$C_{{{\text{Zn}},{\text{mass}}}} = \frac{n \cdot F}{{3.6 \times M}} = 819.9 \;{\text{mAh}}\,{\text{g}}^{ - 1} \approx 820 \;{\text{mAh}}\,{\text{g}}^{{{-}1}}$$

5 $$C_{{{\text{Zn}},{\text{volume}}}} = \frac{{\rho \cdot C_{{{\text{Zn}},{\text{mass}}}} }}{3.6 \times M} = 5853.8 \,{\text{mAh}}\,{\text{cm}}^{ - 3} \approx 5854\, {\text{mAh}}\,{\text{cm}}^{{{-}3}}$$

where *n* represents the number of electrons participating in the redox reaction (*n* = 2 for Zn), *F* is Faraday’s constant (96,485 C mol^−1^), and *M* is the molecular weight in g mol^−1^. The factor 3.6 converts the theoretical specific capacity of C g^−1^ to the more broadly used mAh g^−1^, and *ρ* is the density of Zn (*ρ* = 7.14 g cm^−3^).

These equations indicate that the research strategies employed in previous studies resulted in limited enhancement of the discharge capacity due to the reduction of DOD in thick Zn foils. Thus, a notable improvement in DOD can be achieved by reducing the use of excess Zn. For instance, the DOD for a 100-μm Zn foil only increases slightly from 1.7 to 8.5% upon raising the areal discharge capacity from 1 to 5 mAh cm^−2^. In comparison, the DOD for a 25-μm Zn foil increases significantly from 6.8 to 34.2% with an increase in the areal discharge capacity from 1 to 5 mAh cm^−2^ (Fig. 1c).

The DOD is commonly employed to indicate the Zn utilization in symmetric cells, and the Zn utilization increases with the DOD. In full cells, the Zn utilization is usually increased by reducing the N/P [32, 33]. For instance, under ideal conditions when N/P = 2, the Zn utilization is 50%; when N/P ≈ 1, the Zn utilization can even reach 100% [46]. However, this is not easy to achieve in practical situations, so the Zn utilization for full cells must be reconsidered. The Zn utilization in full cells can be calculated by converting the actual areal capacity of the full cell and the discharge capacity of the anode.

Is it feasible to reduce the amount of excess Zn in the anode or to raise the Zn utilization to approximately 100%? The concept of an anode-free battery was proposed and widely studied in the previous research on lithium metal batteries [47–53]. Inspired by this, anode-free aqueous Zn metal batteries (AF-AZMBs) were also proposed [54]. The AF-AZMBs consist of a Zn-rich cathode as the Zn source and a Zn-free anode with shunned Zn foils or other Zn metal anodes. Zn^2+^ ions are plated in situ on the anode during the first charging process and are fully utilized in the following discharging and charging cycles (Fig. 1d). The deposited Zn metal is the only Zn source available for discharge. This unique Zn-free anode structure gives AF-AZMBs significant advantages over AZMBs: (1) They are more economical. Compared with AZMBs with Zn pre-deposited on the anode, the AF-AZMBs do not require a complex electroplating process to prepare the Zn anode, which reduces the manufacturing cost of the battery. Additionally, the high Zn utilization makes N/P ≈ 1, and the energy and power densities are greatly improved. (2) They are more secure. The Zn metal on the anode current collector is electroplated from the Zn-rich cathode due to the absence of the anode, which means that the assembled AF-AZMBs are fully discharged when stored [54]. This not only avoids self-discharge to increase the capacity, but also makes the storage and operation of the AZMBs safer [55]. (3) They are more accurate. The limited Zn on the cathode side makes the measurement of the CE more accurate. As a result, the electroplating/stripping behavior of Zn on the collector can be evaluated accurately.

**Fig. 2:**
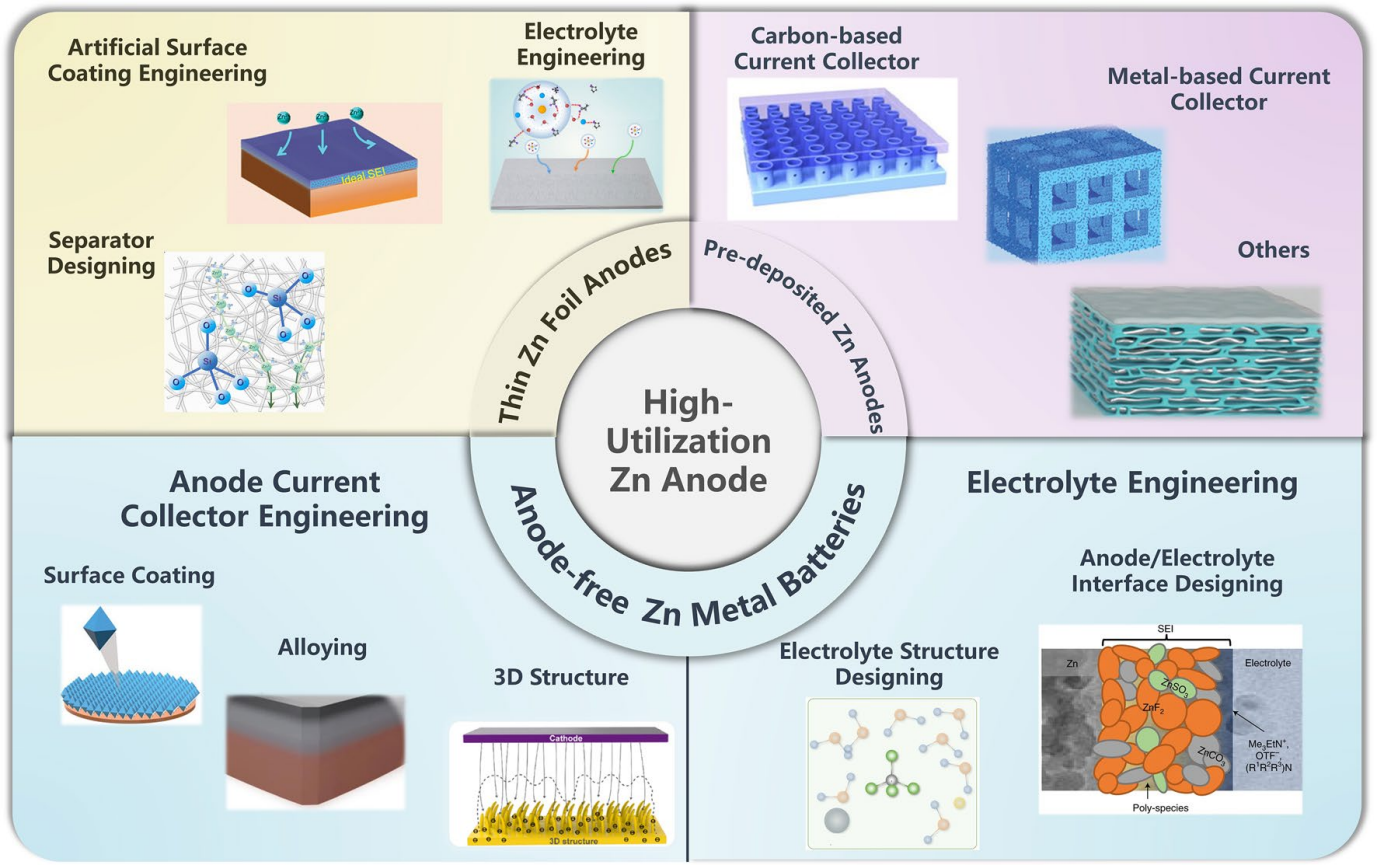
A summary of the design strategies toward the high-utilization Zn anode. Artificial surface coating engineering part: Adapted from Ref. [73]. Copyright 2022, John Wiley and Sons; Electrolyte engineering part: Adapted from Ref. [113]. Copyright 2022, John Wiley and Sons; Separator designing part: Adapted from Ref. [159]. Copyright 2023, Royal Society of Chemistry. Carbon-based current collector part: Adapted from Ref. [173]. Copyright 2023, Elsevier; Metal-based current collector part: Adapted from Ref. [183]. Copyright 2021, John Wiley and Sons; Others part: Adapted from Ref. [191]. Copyright 2021, John Wiley and Sons. Anode current collector engineering of AF-AZMBs part: 3D structure. Adapted from Ref. [197]. Copyright 2022, Elsevier; Surface coating. Adapted from Ref. [201]. Copyright 2023, John Wiley and Sons; Alloying. Adapted from Ref. [203]. Copyright 2023, Open access. Electrolyte engineering of AF-AZMBs part: Anode/electrolyte interface. Adapted from Ref. [214]. Copyright 2021, Springer Nature; Electrolyte structure. Designing. Adapted from Ref. [216]. Copyright 2021, John Wiley and Sons

The methods mentioned above to improve the Zn utilization are gradually being implemented. Nevertheless, how to design AZMBs with high Zn utilization has rarely been systematically discussed and summarized. Herein, we systematically discuss typical strategies for enhancing Zn utilization from the perspective of reducing the amount of excess Zn. First, we summarize the representative methods for calculating the DOD of Zn anodes with different structures. Then, we focus on establishing AZMBs with high Zn utilization by reducing the thickness of Zn foil. Next, we discuss 3D collectors as pre-deposited Zn substrates to reduce the use of excess Zn on the anodes. More importantly, we summarize strategies for constructing stabilized AF-AZMBs with Zn-free anode structures (Fig. 2). Finally, we present the challenges and perspectives for constructing high-Zn-utilization AZMBs with a view to providing comprehensive guidelines for further research.

## Zn Foil Anode

Zn foil is a typical anode material for AZMBs. In previous studies, thick Zn foils (> 100 µm, 58.5 mAh cm^−2^) were commonly used on the anode [56–59]. The excess Zn constantly replenished the active Zn lost during cycling. At a lower areal capacity (≈ 2 mAh cm^−2^), the DOD was only approximately 3.4%, which implied a low utilization of active Zn. When assembled into full cells, the N/P was too high that led to low energy density for full cells. Therefore, overly thick Zn foils constitute a severe impediment for moving AZMBs toward practical application. Currently, a more viable strategy is to reduce the thickness of the Zn foil, but a series of resulting problems must be overcome.

Before using a Zn foil as the anode, the Zn foil surface is made as smooth as possible by polishing, but it is still not flawless. Scratches and pits on the Zn foil surface cause uneven distributions of the electric field and Zn^2+^ ion concentration and promote side reactions and the formation of dendrites, which reinforces the defects on the Zn foil surface and forms a vicious cycle. To make matters worse, thinner Zn foils also cause some new problems. A thin Zn foil is more susceptible to chalking and fragmentation, resulting in a significant loss of active Zn and even cell failure during repeated Zn deposition and stripping, especially with a high Zn utilization. Therefore, for thin Zn foil anodes, strategies such as applying artificial surface coating layers, electrolyte engineering, and separator designing are used to promote uniform Zn deposition and reduce side reactions. The stable Zn foil anodes give the AZMBs with high Zn utilization longer lifespan.

### Artificial Surface Coating Engineering

A series of adverse reactions, such as dendrite growth, HER, corrosion, and passivation, occur at Zn metal anodes during cycling, especially with high Zn utilization. A widely used strategy is to build an artificial protective layer on the anode surface [60–66]. The selection of materials and the design of the protective layer structure are crucial factors, as is the material selected for construction of the artificial protective layer. The widely used protective coating materials include inorganic compounds (such as ZnS [67], ZnF_2_ [68], SiN [69–71], Zn_3_(PO_4_)_2_ [72], and Zn_3_(BO_3_)_2_ [73]), metals and alloys (such as Sn [61], Cd [62], In alloys [74], and Cu/Zn alloys [75]), carbon materials (such as graphene [76], carbon nanotubes (CNTs) [77], carbon cloth [78]and carbon fibers [79]), polymers (such as polyamide (PA) [80]and polyethylene oxide (PEO) [81]), and other materials (such as metal–organic frameworks (MOFs) [82–84], covalent organic frameworks (COFs) [85–87], and MXenes [88]). In the structural design, the artificially coated solid electrolyte interface (SEI) protective layer isolates the interface between the electrolyte and the Zn metal anode and inhibits the HER. Alloying of Zn with other metals by co-deposition effectively adjusts the physical and chemical properties of the Zn metal anode surface and induces heteroepitaxial deposition of Zn. A surface protective layer with a 3D structure can adapt to the volume changes of the Zn electroplating/stripping process, reduce the local current density, and enhance the kinetics of Zn^2+^ ion transfer. An effective combination of different materials and structures can give the Zn anodes with high Zn utilization more significant protection. Table 1 provides a comprehensive summary.

**Table 1:** Summary of electrochemical performance of AZMBs with Zn foil anodes for artificial surface coating engineering in terms of different parameters

Anode/thickness of Zn foil (μm)^a^	Electrolyte^b^	Cathode	Symmetric cells		Full cells		Refs.
			Cycling performance (mAh cm^−2^/lifespan/mA cm^−2^)	Zn utilization^c^	Cycling performance (mAh g^–1^/cycles/A g^–1^ /capacity retention)	N/P	
*SEI*							
ZnS@Zn-350 (10 μm)	1 M ZnSO_4_	MnO_2_	2/1000 h/2	34.2%	110.2/2500/5 C/87.6%	–	[67]
SLM-Zn (7.9 mAh cm^−2^ by pretreatment)	2 M ZnSO_4_	MnO_2_	7.1/180 h/14.2	90%	1.42 mAh cm^−2^/750/0.5/90.6%	4.1	[68]
PSN-Zn (30 μm)	2 M ZnSO_4_	MnO_2_	10/250 h/10	60%	4.22 mAh cm^−2^/400/4/92.4%	4	[69]
Amor-SiN@Zn (30 μm)	2 M ZnSO_4_	NH_4_V_4_O_10_	2/750 h/5	11.4%	144.4/1000/4/89.0%	–	[70]
Zn@NTP (30 μm)	2 M ZnSO_4_	NAVO	10/150 h/10	57%	176/3000/10/–	–	[71]
ZP@Zn (50 μm)	2 M ZnSO_4_	V_6_O_13_	21.74/60 h/26.55	80%	245/1000/2/–	–	[72]
Zn@ZBO (20 μm)	2 M ZnSO_4_	MnO_2_	10/250 h/50	60%	–/2000/10/70%	2.3	[73]
PAZn@Zn (10 μm)	2 M ZnSO_4_	V_2_O_5_	3/200 h/10	51.2%	1.95 mAh cm^−2^/600/2/89.3%	–	[89]
3D Zn@P (50 μm)	3 M ZnSO_4_	NHVO	10/350 h/10	34.2%	107/5000/5/97.3%	–	[90]
MMT-Zn (10 μm)	2 M ZnSO_4_	MMT-MnO_2_	45/1000 h/10	77%	191.5/1100/2/79%	–	[91]
ZnTe@Zn (–)	1 M ZnSO_4_	MnO_2_	1/750 h/30	–	5.85 mAh cm^−2^/500/2 C/–	3 (10 μm)^d^	[92]
SIN_*x*_@Zn (–)	3 M ZnSO_4_	MOV	2/200 h/2	–	–/6000/5/74%	15.8	[93]
Zn@InF_4_ (150 μm)	2 M ZnSO_4_	MnO_2_	10/200 h/10	11.4%	202.3/1000/3/80%	2.8 (10 μm)	[94]
IAZO (100 μm)	2 M ZnSO_4_	MnO_2_@CNT	30/80 h/20	52%	152/1800/1/76%	–	[95]
*Metal and alloy*							
AZO@Zn (10 μm)	2 M ZnSO_4_	V_2_O_5_·H_2_O	4.69/200 h/10	80%	280/500/2/86.8%	–	[60]
Zn/Sn-20 nm (–)	2 M ZnSO_4_	MnO_2_	10/300 h/10	87.8%	115.2/1500/2/78.1%	–	[61]
Zn@Cd (20 μm)	2 M ZnSO_4_	α-MnO_2_	1/3500 h/10	85%	2.0 mAh cm^–2^/100/0.5 C/91.5% (Rest 48 h)	1.7 (10 μm)	[62]
Zn@LM (10 μm)	2 M ZnSO_4_	MnO_2_	1/360 h/1	17%	80/4400/5/–	–	[74]
ILL@Zn (20 μm)	2 M ZnSO_4_	NMO	10/250 h/20	85%	96.39/1500/1/82.6%	–	[75]
HP-Zn (100 μm)	2 M ZnSO_4_	ZVO	20/240 h/1	35%	–/300/5/85.03%	5.5 (10 μm)	[96]
*Carbon*							
NGO@Zn (100 μm)	1 M ZnSO_4_	LMO	5/300 h/5	8.5%	163 Wh kg^–1^/178/1/80%	–	[76]
CNTguard-Zn (25 μm)	2 M ZnSO_4_	AC	14/100 h/2	97%	–/10000/50 mA cm^−2^/92%	–	[77]
Zn@MFCs (100 μm)	2 M ZnSO_4_	α-MnO_2_	10/200 h/10	17%	195.5/600/1/82.8%	–	[78]
NCL-Zn (50 μm)	2 M ZnSO_4_	MnO_2_	1/4000 h/1	38%	–/1000/1/93.58%	–	[79]
FPCH-ZI/Zn (10 μm)	2 M ZnSO_4_	MnO_2_	3/110 h/3	51%	–/1000/0.5/–	7.3 (10 μm)	[97]
*Polymer*							
Coated Zn (20 μm)	2 M ZnSO_4_	MnO_2_	10/75 h/10	85%	–/1000/2/88%	–	[80]
Zn-PG (10 μm)	3 M Zn(OTf)_2_	NH_4_V_4_O_10_	10.2/1000 h/5	90%	–/2000/5/30%	–	[81]
60 Alucone@Zn (–)	3 M Zn(CF_3_SO_3_)_2_	MnO_2_	10/300 h/1	8.5%	208.9/800/1/83.3%	–	[100]
Zn (PVA@SR-ZnMoO_4_) (50 μm)	3 M ZnSO_4_	α-MnO_2_	10/250 h/10	34%	141.7/1000/1/59.1%	–	[101]
Zn@NH_2_-PSiO_*x*_ (30 μm)	2 M ZnSO_4_	MnO_2_	10/300 h/20	57%	2.93 mAh cm^–2^/100/0.5 C/–	1.8	[102]
PILZ@Zn (40 μm)	1 M ZnSO_4_	MnO_2_	1/2000 h/1	4.3%	–/100/0.1/91%	–	[103]
Zn-PZIL (–)	2 M ZnSO_4_	MnO_2_	10/350 h/40	74.3%	146/2000/10 C/–	–	[104]
*MXenes/MOFs/COFs*							
Zn (–)	Zn(TFSI)_2_-TFEP@MOF/H_2_O	MnO_2_	0.5/700 h/0.5	–	135/600/10 C/97.2%	2	[82]
Zn@600 nm-ZIF-8 (100 μm)	2 M ZnSO_4_	MnO_2_	50/90 h/50	85%	–/1000/1/100%	–	[83]
FCOF@Zn (–)	2 M ZnSO_4_	MnO_2_	1/1700 h/5	–	–/250/3 mA cm^−2^/–	2	[87]
CNF/MXene@Zn (100 μm)	3 M Zn(CF_3_SO_3_)_2_	VO_2_	100/120 h/100	88.2%	–/100/1/86.2%	2.8 (10 μm)	[88]
ZGL@Zn (20 μm)	2 M ZnSO_4_	MnO_2_	10/250 h/1	85.5%	–/1200/1/98.2%	–	[89]
MX-TMA@Zn (20 μm)	2 M ZnSO_4_	MnO_2_Ti_3_C_2_	10/450 h/10	85%	251.7/2000/2/–	–	[105]

^a^Thickness of Zn foil for symmetric cells

^b^Electrolyte of symmetric cells

^c^The Zn utilization is calculated using Eq. (2)

^d^Thickness of Zn foil for full cells

**Fig. 3:**
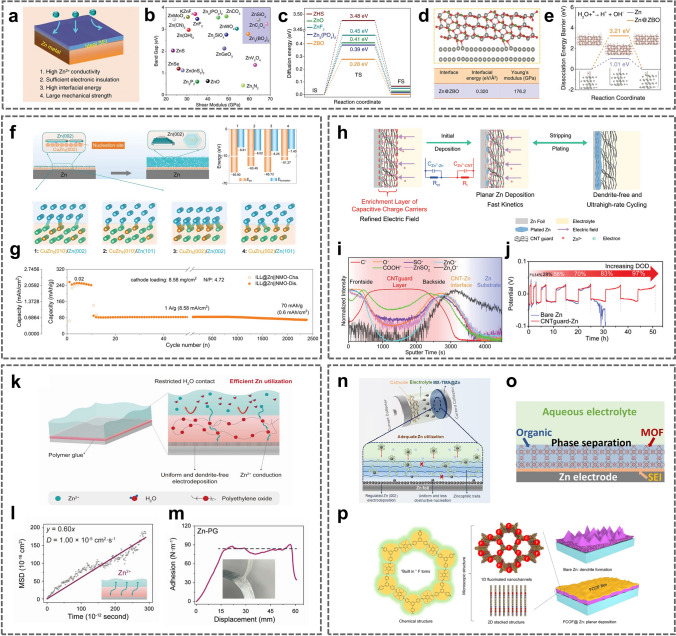
**a** The pivotal elements for ideal SEI materials on the Zn surface. **b** Bandgaps and shear moduli of potential SEI candidates. **c** Energy barriers of Zn^2+^ diffusion on ZHS, ZnO, ZnF_2_, Zn_3_(PO_4_)_2_, and ZBO. **d** Interface structure, interfacial energy (*γ*), and Young’s modulus (*E*) of Zn@ZBO. **e** The dissociation energy barriers of H_2_O on the bare Zn and Zn@ZBO. Adapted from Ref. [73]. Copyright 2022, John Wiley and Sons. **f** Calculation models and calculated total system energies (*E*_tot_) and formation energies (*E*_formation_) between CuZn_5_ (CuZn_5_(010), CuZn_5_(002)) and Zn (Zn(002), Zn(101)) at the ZEI. **g** Cycling performance of ILL@Zn||NMO full cell with a low N/P (4.72). Adapted from Ref. [75]. Copyright 2023, John Wiley and Sons. **h** Schematic illustration of dendrite-free Zn deposition on CNT_guard-_Zn during the Zn stripping/plating. **i** Normalized intensity of C^−^, O^−^, COOH^−^, SO^−^, ZnSO^2−^, ZnO^−^, and Zn_3_O^−^ in CNT_guard_–Zn after cycling along the sputtering time from TOF–SIMS. **j** The cycling performance of the CNT_guard_–Zn symmetric cell. Adapted from Ref. [77]. Copyright 2023, John Wiley and Sons. **k** Schematic illustration of an ion-selective polymer glue coated on Zn anode. **l** Simulated mean square displacement (denoted as MSD) of Zn^2+^ in polymer glue as a function of simulated time. **m** Pull-off adhesion test of Zn foil and polymer glue. Adapted from Ref. [81]. Copyright 2021, John Wiley and Sons. **n** Schematic diagram of Zn deposition behavior on MX-TMA@Zn. Adapted from Ref. [105]. Copyright 2022, Elsevier. **o** SEI formation in MOF confined organic electrolyte. Adapted from Ref. [82]. Copyright 2020, John Wiley and Sons. **p** The structure and working mechanism of the FCOF film. Adapted from Ref. [87]. Copyright 2021, Open access

The construction of a strong and dense SEI on the Zn metal anode surface with inorganic compounds is an efficient strategy [67–72, 89–95]. Generally, an ideal SEI layer should meet the following characteristics. First, the coating should possess high Zn^2+^ ionic conductivity and electrical insulation to allow Zn deposition at the interface between the coating and anode. Second, the coating should be dense and stable in the electrolyte to prevent direct contact between the electrolyte and the metal anode surface. In addition, good mechanical properties and close bonding with the metal anode surface should also be considered so the coating plays a stable protective role during long-term cycling. Yang et al. proposed a method for screening potential SEIs on anodes (Fig. 3a) [73]. The charge transfer capability of different SEI materials was evaluated from the band gaps of the materials and the potential barriers for Zn^2+^ ion diffusion (Fig. 3b, c). The dendrite suppression capability was determined from the interfacial energy (*γ*) and Young’s modulus (*E*), *γE* (Fig. 3d). After a comprehensive comparison, they identified Zn_3_(BO_3_)_2_ (ZBO) as one of the most promising candidates that effectively promoted the uniform deposition and lateral growth of Zn. In addition, the high dissociation energy barrier for H_2_O on Zn@ZBO effectively inhibited side reactions (Fig. 3e). As a result, a symmetric Zn cell using Zn@ZBO was cycled stably for 250 h (50 mA cm^−2^, 10 mAh cm^−2^) with up to a 60% Zn utilization.

An advantage of constructing a metal or alloy protective layer is that it induces Zn deposition along Zn (002) plane, which causes the Zn to grow epitaxially layer by layer [74, 96]. It effectively inhibits the overgrowth of Zn dendrites, and Zn (002) planes possess a better corrosion resistant. A common approach is to construct an interfacial protective layer with a low degree of Zn lattice mismatch. In the first stage of Zn plating, the low mismatch crystalline surfaces of the protective layer provide heterogeneous nucleation and guide nucleation and epitaxial growth of Zn (002) crystalline surfaces. Finally, a uniform and dense Zn deposition layer is obtained. Huang et al. prepared a Cu–Zn alloy lattice interface-locked layer (ILL) by co-electrodeposition [75]. The ILL had a low lattice mismatch with Zn (*δ* = 0.036). And there is high total interfacial energy and formation energy between CuZn_5_ (002) and Zn (002). Therefore, the ILL can as a interfacial lattice locking layer for planar and stable Zn deposition (Fig. 3f). With a limited Zn supply (N/P = 4.72), the ILL@ZN||NMO cell was stably cycled for 2300 h (Fig. 3g).

Carbon materials are also used extensively as protective layers [78, 79, 97]. Graphene exhibits a slight lattice mismatch with Zn (002) and possesses a low binding force with Zn [98]. Chen et al. synthesized an artificial interface film comprising nitrogen (N)-doped graphene oxide (NGO) to provide a parallel and ultrathin interface modification layer (≈ 120 nm) on the Zn foil [76]. The pyrrolic nitrogen-containing functional group resulted in a high binding energy between the NGO and Zn, which enhanced the ability of Zn trapping by the NGO. The uniform electric field and zincophilic sites induced uniform distribution/adsorption of Zn^2+^ ions, resulting in a flat deposition morphology and stable interface at the NGO@Zn electrode. 3D structured carbon nanotubes (CNTs) have also attracted attention because of their ability to reduce the local current density and induce uniform Zn nucleation and growth. Li et al. used zincophilic CNTs as a protective layer for the Zn foil anode (Fig. 3h) [77]. After hydrophilization, the CNTs exhibited good wettability with the electrolyte and transported Zn^2+^ ions efficiently. They found that the CNTs were capacitive before Zn reduction at the CNTs–Zn interface. This resulted in enrichment of Zn^2+^ ions and electrons at the interface, which generated higher electrochemical activity at the CNT–Zn interface. The species distributions identified with time-of-flight secondary ion mass spectrometry (TOF–SIMS) indicated that Zn deposition occurred mainly at the CNTs–Zn interface (Fig. 3i). Based on these advantages, the CNT_guard_–Zn symmetric cells exhibited sustain good stability ranging from 2 to 97% DOD (Fig. 3j).

When constructing a hydrogel protective layer, the requirement is close bonding of the hydrogel protective layer with the Zn anode and rapid diffusion of Zn^2+^ ions within the gel layer [80, 99–104]. Zhang et al. designed a new ion-selective polymer gel as a protective layer for Zn metal anodes (Fig. 3k) [81]. The hydrogel layer provided fast Zn^2+^ ion migration with a high diffusion coefficient, showing fast Zn stripping/plating kinetics (Fig. 3l). Additionally, diffusion of water in the hydrogel was limited, which prevented direct contact of the water with the new metal anodes surface. Meanwhile, the hydrogel layer was effectively bonded to the Zn foil, resulting in a high diffusion energy barrier for lateral diffusion of Zn^2+^ ions at the interface and facilitating dendrite-free Zn deposition (Fig. 3m). In addition, isolating the electrode from H_2_O with the tightly bound gel interface layer effectively suppressed side reactions such as corrosion and passivation. On this basis, the hydrogel-protected Zn metal anode achieved a high Zn utilization of 90% at a high current density of 5 mA cm^−2^ for an extremely long period of 1000 h. The full cell exhibited high energy and power densities with a long lifetime. At high Zn utilization of 50%, the full cell exhibited high specific capacity of 410 mAh g^−1^ based on the effective mass of the cathode.

MXenes, MOFs and COFs have also been utilized as protective layers on Zn metal anodes [82–88, 105, 106]. Ye et al. constructed a tetramethylammonium-intercalated Ti_3_C_2_T_*x*_ MXene (MX-TMA) coating with a low Zn nucleation barrier on the surface of Zn foil (Fig. 3n) [105]. The coating showed high hydrophilicity and abundant zincophilic sites for the protective layer. Moreover, the MX-TMA guided directional and homogeneous deposition of Zn on the beneficial (002) planes by modulating the synergistic effect of zincophilic sites and a low lattice match (~ 10%) between the MXene and Zn. As a result, the lifespan of the Zn anode was extended by 3600 h at 2 mA cm^−2^. Even under deep discharge (DOD_Zn_ ≈ 85%), the MX-TMA@Zn anode still operated stably more than 450 h. Wang et al. utilized the MOF-encapsulated Zn(TFSI)_2_-tris(2,2,2-trifluoroethyl)phosphate (TFEP) electrolyte to form a ZnF_2_-Zn_3_(PO_4_)_2_ SEI to inhibit Zn dendrites and HER (Fig. 3o) [82]. With the capacity ratio of Zn:MnO_2_ at 2:1, the full cell in Zn(TFSI)_2_-TFEP@MOF/H_2_O electrolyte still maintained 141 mAh g^−1^ with CE approaching 100% after 600 cycles. Lu et al. constructed an ultrathin, fluorinated two-dimensional porous covalent organic framework (FCOF) film as a protective layer on the Zn anode surface (Fig. 3p) [87]. The strong interaction between fluorine (F) in FCOF and Zn reduced the surface energy of the Zn (002) crystal plane, enabling horizontal parallel Zn deposition. Thus, the FCOF@Zn/MnO_2_ full cell with N/P = 5:1 exhibited a stable lifespan with more than 200 cycles even at 4 mA cm^−2^.

The artificial SEI layers introduced above can protect Zn anode and guide uniform Zn^2+^ ion plating/stripping, but their further application is hampered by some issues, such as tedious preparation process and poor compatibility among anode, protective coating and electrolyte. When Zn is deposited on the metal surface with good zincophilicity such as Ag and Cu, Zn can enter the metal lattices to form alloys, which improves the substrate zincophilicity to promote the uniform Zn plating/stripping. However, when discussing the zincophilicity of the metal, only the Zn deposition process on metal surface is considered, while the influence of the metal/alloy in the stripping process is ignored. Carbon-based coating layers play a key role in the stability of Zn anodes. This is because the porous structure can provide sufficient space for Zn deposition and homogenize the ion diffusion to avoid “tip effect”. Moreover, the good mechanical strength of carbon-based coating layers can further inhibit the dendrite growth. However, due to the conductivity of carbon materials and/or the zincophilic sites provided by heteroatoms, Zn is preferentially deposited onto the protective layer, resulting in the protection failure of the carbon-based coating layers. MOFs and COFs possess many advantages, such as high porosity, low density, large surface area, strong skeleton, adjustable porous morphology, and rich physical and chemical characteristics. Therefore, as the artificial coating layers of Zn anodes, MOFs and COFs can be used as the diffusion channels or enrichment deposition centers of metal ions to adjust the uniform Zn nucleation, guide the uniform Zn deposition, reduce the polarization voltage, and realize dendrite inhibition. Nevertheless, the further application of MOFs and COFs in AZMBs is limited by the complicated synthesis process and high cost. MXenes with 2D structure exhibit high electronic conductivity, low ion diffusion barrier and good mechanical properties. Abundant functional groups of MXenes can provide strong binding for Zn and serve as nucleation sites to induce uniform Zn nucleation and deposition. However, MXenes are easily oxidized due to the presence of many defects/vacancies and exposed metal atoms/clusters on the surface, thus deteriorating the inherent properties. Therefore, improving the chemical stability of MXenes is a critical task for practical applications. Hydrogels are constructed of saturated water and cross-linked polymer networks, which results in hydrogels and aqueous batteries being inherently compatible. The hydrogel elastomer formed by the polymer networks can promote the device flexibility, and the hydrogel anionic conductor formed by the water molecules can ensure the ion transport during charge/discharge. However, the weak ionic conductivity also brings other issues to the Zn anode. For instance, the dynamics of ion transfer may be slowed due to increased polarization.

In conclusion, the construction of artificial protective layer is an effective strategy to improve the Zn utilization and electrochemical performance of AZMBs. However, in selecting and designing protective layer materials and structures, the electrochemical performance, chemical stability, preparation process, and other factors must be considered wholistically to achieve a better protection effect.

### Electrolyte Engineering

The electrolyte influences the diffusion of Zn^2+^ ions and the electrochemical reactions occurring at the electrode/electrolyte interface, which determines the electrochemical performance of AZMBs. Undesirable reactions at the electrode/electrolyte interface, such as dendrite growth, HER and corrosion, hinder the construction of Zn metal anodes with high Zn utilization [107–109]. In order to alleviate the above problems and improve the Zn utilization, electrolyte engineering mainly focuses on utilizing hybrid electrolytes, electrolyte additives, and novel electrolyte systems such as gel electrolytes and solid electrolytes. The hybrid electrolytes can effectively reduce the free water activity, break the hydrogen-bond network and weaken the Zn^2+^ solvation [110–116]. The electrolyte additives can reshape the Zn^2+^ solvation sheaths [117, 118], modulate the electric double layers (EDL) [119], or form protective in situ SEI layers at electrode/electrolyte interface [120]. Polymer hydrogel electrolytes possess less free water and better electrochemical stability than aqueous electrolytes, as well as good mechanical properties and flexibility required to form the reasonable interface. Table 2 provides a comprehensive summary.

**Table 2:** Summary of electrochemical performance of AZMBs with Zn foil anodes for electrolyte engineering in terms of different parameters

Anode/thickness of Zn foil (μm)^a^	Electrolyte^b^	Cathode	Symmetric cells		Full cells		Refs.
			Cycling performance (mAh cm^−2^/lifespan/mA cm^−2^)	Zn utilization^c^	Cycling performance (mAh g^−1^/cycles/A g^−1^/capacity retention)	N/P	
*Solvation sheaths*							
Zn (165 μm)	Zn(DBS)_2_	Na_3_V_2_(PO_4_)_3_	0.5/2000 h/– (Asymmetric cells)	50%	–/105/0.3 mA cm^−2^ /79.7%	2 (10 μm)^d^	[110]
Zn (10 μm)	Anti-M-50%	PANI	0.5/900 cycles/1 (Asymmetric cells)	99.7% (CE)	–/2000/5/89.3%	–	[111]
Zn (10 μm)	50% H_2_O + 50% DME + 50 mM I^–^	VS_2_@SS	13.35/300 h/8.85	75.5%	–/160/0.1/83.9%	3.64	[112]
Zn (8 μm)	25% SL	V_2_O_5_	24/110 h/24	96%	–/500/10/70%	1.5	[113]
Zn (4 mA h cm^–2^)	1 M ZnSO_4_ + HWAE-10	MnO_2_	2/600 h/2	50%	–/100/0.3/90%	10 (Pre-deposited Zn on Cu foil)	[117]
Zn (–)	2MG1	MnO_2_	10/1170 h/10	–	287/400/0.5/87%	–	[118]
Zn (30 μm)	3 M Zn (OTF)_2_-(H_2_O-HAc)	V_2_O_5_	5/700 h/1	28.4%	129.63/3000/10 C /89.4%	2.2	[121]
Zn (10 μm)	Zn(OTF)_2_ + TMU	V_2_O_5_	5/230 h/5	86%	–/2000/1/–	3.3	[122]
Zn (100 μm)	ZnSO_4_-7H_2_O-3DMF	AC	1/1100 h/3	1.7%	–/5000/0.5/80.6%	4 (20 μm)	[123]
Zn (30 μm)	RME	Zn_0.25_V_2_O_5_·nH_2_O	5/1100 h/5	31.2%	–/390/1.1 mA cm^–2^ /80%	3.2	[124]
*Interface*							
Zn (100 μm)	4 M Zn(TFSI)_2_ + 4 M P_444(2O1)_-TFSI	HNVO	2.5/250 h/2.5	4.3%	–/80/–/95%	6 (10 μm)	[119]
Zn (70 μm)	Sac/ZnSO_4_	MnO_2_	10/500 h/10	24.4%	100 mAh g^–1^ /7500/2.4/–	–	[125]
Zn (10 μm)	ZSO-2959	VS_2_	1/3800 h/1	17%	–/700/0.5/97.8%	2.5	[126]
Zn (50 μm)	Zn(TFSI)_2_ + ZIF-90-I	MnO_2_	1/1200 h/5	3.4%	–/3659/2/–	–	[127]
Zn (20 μm)	2 M ZnSO_4_ + 0.1 M MSG	NH_4_V_4_O_10_	9.11/450 h/9.11	80%	183.4/1000/2/–	–	[128]
Zn (10 μm)	0.1 wt% of N, S-CDs + 2 M ZnSO_4_	Na_2_V_6_O_16_·3H_2_O	7.8/200 h/3.9	67%	–/90/1/91.2%	1.05 (10 μm)	[129]
Zn (20 μm)	TCNQ	β-MnO_2_	5/220 h/5	43%	143.3/4000/2/94.7%	–	[130]
Zn (20 μm)	2 M Zn(OTf)_2_ + 30% TMS	PANI	1/600 h/2	26%	56.7/2000/2 mA cm^−2^ /100%	–	[131]
Zn (15 μm)	ZnCl_2_/H_2_O-40GBL	I_2_@TCC	2/600 h/10	22.78%	–/650/2/77.2%	50% DOD (Zn@Cu, 3.0 mAh cm^–2^)	[132]
Zn (13 μm)	0.0085 M La(NO_3_)_3_ + 2 M ZnSO_4_	VS_2_	5.93/160 h/10	80%	1.33 mAh cm^–2^ /1000/16.0 mA cm^−2^/–	–	[133]
Zn (100 μm)	ZnSO_4_-ace	MnO_2_	50/800 h/50	73.5%	170/1000/1/98.8%	–	[134]
Zn (100 μm)	2 M ZnCl_2_ + 0.4 M NaI	VO_2_	30/150/30	51.3%	–/300/1/75.7%	–	[135]
Zn (20 μm)	3D Zn in TBA	α-MnO_2_	5/160 h/5	42.7%	–/300/1/–	–	[136]
Zn (50 μm)	β-CD/ZnSO_4_	V_2_O_5_	20/200 h/20	68.3%	–/1000/6/71%	–	[137]
Zn (40 μm)	ImS/ZSO	NVOH	20/350 h/10	85%	–/1500/5/91.6%	–	[138]
*SEI*							
Zn (–)	2 M ZnSO_4_ + DFA	MnO_2_	1/1100 h/1	–	170/1000/0.5/–	1.3 (10 μm)	[108]
Zn (250 μm)	2 M ZnSO_4_-50 mM DOTf	Zn_0.25_V_2_O_5_·nH_2_O	4/350 h/4	2.7%	–/1000/–/83%	2.5	[109]
Zn (30 μm)	2 M ZnSO_4_ + 0.5 M Zn(OTF)_2_	MnO_2_	10/275 h/10	62%	208/1000/1/–	–	[114]
Zn (20 μm)	ZnSO_4_-H_2_O-NMP	MnO_2_	10/200 h/5	85.6%	–/150/0.5 C/87.7%	2.3 (20 μm)	[120]
Zn (20 μm)	SN	MVO	10/325 h/2	86.1%	–/3250/20/–	–	[139]
Zn (20 μm)	Zn(OTF)_2_ HMPA H_2_O	V_2_O_5_	10/200 h/40	85.6%	–/400/1 mA cm^−2^/–	1.8 (20 μm)	[140]
Zn (100 μm)	2 M ZnSO_4_ + 2 mM SeO_2_	MnO_2_	2/2000 h/2	3.4%	–/60/0.2/105.6%	4.2 (10 μm)	[141]
Zn (100 μm)	PEGTE-5	V_2_O_5_	10/1300 h/10	17%	–/600/1/–	3 (10 μm)	[142]
Zn (10 mAh)	Zn(NH_2_SO_3_)_2_	O_d_-NVO	7/250 h/7	70%	–/5000/5/95%	(A Ti plate pre-deposited with 10 mAh Zn)	[143]
Zn (10 μm)	2 M ZnSO_4_ + 0.05 M SG	PANI	5/100 h/5	85%	–/1400/5/81.7%	–	[144]
Zn (30 μm)	CO_2_ in the ZnSO_4_	V_2_O_5_	10/100 h/10	57%	–/1000/5/66%	–	[145]
Zn (50 μm)	2 M ZnSO_4_ + 0.02 M EMImOTF	V_6_O_13_	20/6600 h/20	68%	–/350/3.5 mA cm^−2^ /100%	1.73 (Deposited Zn on the Ti net)	[147]
Zn (20 μm)	10% PEGDA	ZnVO	11.71/300 h/7.5	64%	–/2500/5/–	6.4	[115]
Zn (100 μm)	ZCE–2	AC	50/2000 h/50	85%	54.2/3000/5/–	–	[148]
Zn (60 μm)	1 M Zn(CF_3_SO_3_)_2_ + 25 mM Zn(H_2_PO_4_)_2_	V_2_O_5_	5/800 h/1	14.2%	–/500/–/94.4%	2.3 (10 μm)	[149]
*Hydrogel electrolytes*							
Zn (–)	colloid-polymer electrolyte	Na_5_V_12_O_32_	20/100 cycles/100 (Asymmetric cells)	–	–/120/5/84.8%	1 (Pre-deposited Zn on Cu)	[107]
Zn (200 μm)	PCZ-gel	NH_4_V_4_O_10_	9.11/440 h/9.11	80%	195/2000/2/86%	–	[150]
Zn (15 μm)	SFPAM-Zr	PANI	5/200 h/5	57%	85/5000/5/–	–	[151]
Zn (100 μm)	CarraChi gel	ZVO	35/4000 h/10	65%	0.9 Ah/200/0.2/84%	–	[152]
Zn (80 μm)	In situ GPE	ZHC	40/240 h/40	87%	–/8000/10/88%	–	[153]

^a^Thickness of Zn foil (μm) for symmetric cells

^b^Electrolyte of symmetric cells

^c^The Zn utilization is calculated using Eq. (2)

^d^Thickness of Zn foil for full cells

**Fig. 4:**
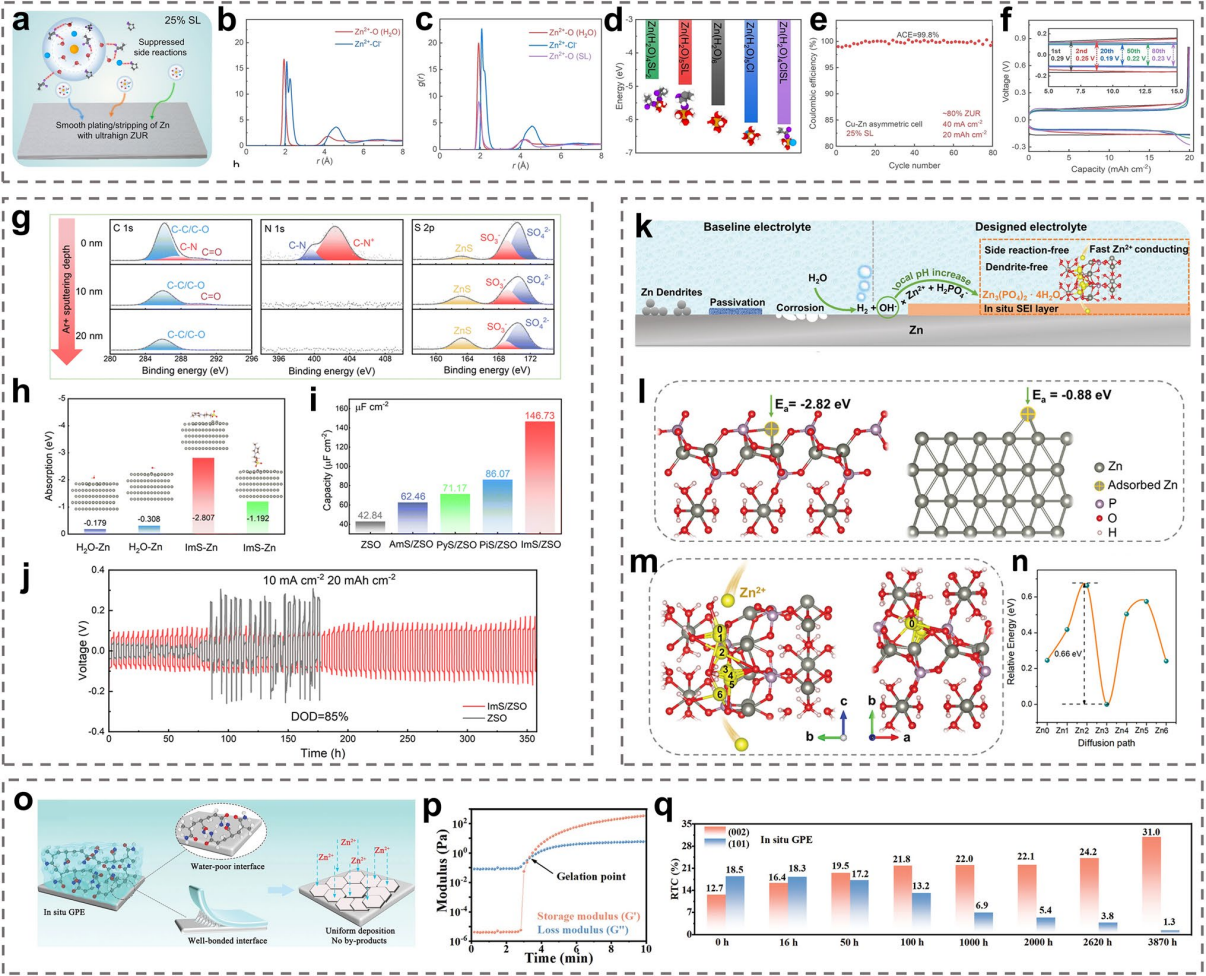
**a** Schematic diagram of the solvation structures of Zn^2+^ in the electrolytes with 25% sulfolane (SL) and the interfacial reactions. Radial distribution functions (RDFs) of **b** 0% SL, and **c** 25% SL electrolytes. **d** DFT calculation for binding energies of different solvation structures in the electrolyte with 25% SL. **e** CE and **f** voltage curves of plating/stripping in the electrolyte with 25% SL at 40 mA cm^−2^ and 20 mAh cm^−2^ (~ 80% Zn utilization). Adapted from Ref. [113]. Copyright 2022, John Wiley and Sons. **g** XPS depth profiles of the Zn anode surface after 100 h. **h** Adsorption energies of the water molecule and ImS on the Zn metal anode. **i** Double layer capacitance at electrode/electrolyte interfaces in ZSO, AmS/ZSO, PyS/ZSO, PiS/ZSO, and ImS/ZSO. **j** Cycling performances of Zn||Zn symmetric cells. Adapted from Ref. [138]. Copyright 2022, Royal Society of Chemistry. **k** Schematic diagram of the evolution on the Zn surface and the mechanism of the SEI formation. **l** Calculated models of the interaction between the absorbed Zn^2+^ and the surfaces of the SEI layer and the bare Zn. **m** The optimum Zn^2+^ diffusion pathway in the SEI layer, and **n** the corresponding migration energy barrier. Adapted from Ref. [149]. Copyright 2021, John Wiley and Sons. **o** Schematic diagram of in situ gel polymer electrolytes (GPE) at the electrode/electrolyte interface. **p** Storage modulus (*G*′) and loss modulus (*G*″) of in situ GPE as functions of time. **q** Calculated the relative texture coefficients (RTCs) of different crystal planes with in situ GPE. Adapted from Ref. [153]. Copyright 2022, John Wiley and Sons

During electrodeposition process, the Zn^2+^ ions form tight solvation sheaths ([Zn(H_2_O)_6_]^2+^) with six free H_2_O molecules and provide many reactive H_2_O molecules at the anode/electrolyte interface, which leads to various side reactions, such as HER [111]. In addition, the increase of local pH caused by H_2_O decomposition accelerates the formation of byproducts [112, 121]. Hybrid electrolytes and electrolyte additives can improve the reversibility of Zn metal anodes by modulating the solvation configuration around Zn^2+^ ions to inhibit Zn dendrites and side reactions [115, 116, 122–124]. Chen et al. added sulfolane (SL) to the electrolyte [113]. The SL changed the primary solvation structure of Zn^2+^ ions, effectively inhibiting the activity of H_2_O from the aqueous solution and significantly improving the electrochemical stability of Zn metal anodes (Fig. 4a–c). With the addition of SL, the most stable structure in the electrolyte was Zn(H_2_O)_4_ClSL, and an overly high concentration caused displacement of the SL by water molecules (Fig. 4d). With a 25% concentration of SL, the resulting Zn||Zn symmetric cell was stable for more than 80 cycles at a high Zn utilization of 80% (20 mAh cm^−2^) and 40 mA cm^−2^ with the average CE of 99.8% and the overpotential of 0.19 V (Fig. 4e, f). With N/P = 1.5, the Zn||V_2_O_5_ full cell was stable for 500 cycles at 10 A g^−1^, with a capacity retention of 70% and Zn utilization of up to 67%.

The electric double layer (EDL) influences the electrochemical processes occurring at the interface, such as desolvation and reduction of Zn^2+^ ions and the formation of SEI films [125–127]. Zn^2+^ ions and many free water molecules are tightly adsorbed on the Zn metal surface with negative charge [119, 128]. Electrostatic repulsion and shielding effects cause the solubilized Zn^2+^ ions to be dispersed in the diffusion layer [129–131]. Direct contact between the free water and Zn metal leads to HER and triggers chain reactions such as corrosion and passivation [132]. Electrolyte additives can tune the structure of the EDL precisely. The addition of high valent ions can reduce the double layer thickness and double layer repulsion, thus enabling dense and homogeneous Zn deposition [133, 134]. Additionally, competing ions can be introduced for preferential adsorption at the anode surface, so that the direct contact between water molecules and the Zn metal surface can be inhibited [135]. The electrostatic shield can prevent the “tip effect” and inhibit dendrite formation [136, 137]. Chen et al. added the amphoteric ionic liquid (ZIL) to the electrolyte, and 3-(1-methylimidazole) propane sulfonate (ImS) was stably adsorbed on the electrode surface (Fig. 4g) [138]. The strong adsorption of ImS on the Zn metal anode caused the ImS to enrich and preferentially occupy the highly active sites on the Zn metal surface, forming a dynamic electrostatic shielding layer and a unique water-poor interface on the Zn anode (Fig. 4h, i). The dynamic electrostatic shield layer inhibited deposition of Zn^2+^ at the high-potential protrusions. It also ensured 3D diffusion of Zn^2+^ ions and suppressed 2D diffusion in the adapted EDL. Formation of the water-poor interface was attributed to interaction of the ImS additive with water to release numerous active sites, thereby limiting occupation of the active sites on the surface by active water molecules. Therefore, even at a high current density (10 mA cm^−2^), the anode surface still exhibited a uniform and dense Zn deposition morphology, with Zn utilization as high as 85% (20 mAh cm^−2^) (Fig. 4j).

Although preparing SEI layers formed ex situ by chemical deposition or physical coating on a Zn foil surface is complex and time-consuming, the SEI layers formed in situ by adding electrolyte additives or utilizing hybrid electrolytes are more useful for practical application, especially when high Zn utilization is desired [108, 109, 114, 115, 120, 139–148]. Guo et al. constructed dense and stable SEI layers in AZMBs in situ by introducing Zn(H_2_PO_4_)_2_ into the electrolyte (Fig. 4k) [149]. The formation mechanism is as follows:

6 $$2{\text{H}}_{2} {\text{O}} + 2e^{ - } \to {\text{H}}_{2} \uparrow + 2{\text{OH}}^{ - }$$

7 $$2{\text{H}}_{2} {\text{PO}}_{4}^{ - } + 4{\text{OH}}^{ - } + 3{\text{Zn}}^{2 + } \to {\text{Zn}}_{3} \left( {{\text{PO}}_{4} } \right)_{2} \cdot 4{\text{H}}_{2} {\text{O}} \downarrow$$

The SEI layer with a thickness of approximately 140 nm possessed a high adsorption capacity for Zn^2+^ ions, which led to a uniform Zn^2+^ ion flux at the Zn metal surface and promoted uniform Zn deposition (Fig. 4l). In addition, the Zn^2+^ ion diffusion channels in the SEI corresponded to low Zn^2+^ ion diffusion energy barriers, ensuring uniform and efficient Zn^2+^ ion diffusion, suppression of electrolyte-induced side reactions and a dendrite-free, uniform Zn deposition morphology, thus improving the stability and reversibility of the Zn metal anode (Fig. 4m, n).

The abundant hydrophilic groups of the gel electrolyte can adsorb considerable water molecules, giving the gel electrolyte good ionic conductivity [107, 150]. Since the gel electrolyte generally possesses the good mechanical strength and flexibility, its role is similar to that of a battery separator [151]. In addition, the reduced free water content can effectively inhibit the HER, corrosion, passivation, and by-product formation at the electrolyte/electrode interface [152]. However, ex situ prepared gel electrolytes often require more contact with the electrode interface due to the increased interfacial impedance and gradual degradation of the interface environment during long cycling, which is caused by dendrite growth and volume changes. The intrinsic reducing capacity of Zn can be used to prepare gel electrolytes in situ [153]. The reduction–oxidation reaction between Zn metal and potassium persulfate (KPS) effectively generated SO_4_^–^ radical ions, which in turn triggered the polymerization of acrylamide (AM) in the precursor solution (Fig. 4o).

8 $$0.5{\text{Zn}} + {\text{S}}_{2} {\text{O}}_{8}^{2 - } \to 0.5{\text{Zn}}^{2 + } + {\text{SO}}_{4}^{{2{-}}} + {\text{SO}}_{4}^{{\cdot{-}}}$$

The storage and loss moduli of the in situ gel electrolyte led to rapid polymerization of the precursor solution at the Zn foil within 3 min (Fig. 4p). The in situ welded gel electrolyte exhibited good interfacial contact and strong bonding to the Zn metal anode. It effectively inhibited side reactions at the electrode/electrolyte interface and prevented 2D diffusion of Zn^2+^ ions on the anode surface. Changes in the relative transfer coefficients (RTCs) of the (101) and (002) planes showed that the solid chemically bonded interface modulated the deposition of Zn along the (002) plane, which had a lower surface energy (Fig. 4q). These properties of the in situ gel electrolyte allowed the stacked cell to achieve a stable lifespan of 240 h even at a high current density (40 mA cm^−2^) and DOD (40 mAh cm^−2^, ≈ 87%). The in situ gel electrolyte mitigated interfacial problems such as dendrite growth and interfacial side reactions through adequate contact with the solid electrode interface.

In summary, Zn anodes are effectively regulated through optimizing electrolytes to achieve dendrite-free growth and inhibit side reactions. However, when electrolyte engineering optimizes anode/electrolyte interface and changes solvation structure of Zn^2+^ ions, it also affects the cathode structure and cathode/electrolyte interface property. Therefore, designing electrolytes with more comprehensive functions is an important direction for the development of electrolyte engineering. Moreover, it is necessary to develop electrolyte optimization strategies with high Zn utilization and lean electrolytes for commercialization of AZMBs. Under lean electrolyte conditions, designing the electrolyte with good stability can reduce the sharp decrease in performance caused by the electrolyte decomposition.

### Separator Designing

As a vital component of the battery system, the separator stores electrolytes and provides channels to connect the anode and cathode while physically isolating the anode and cathode to prevent short circuits [154, 155]. In AZMBs, the requirements of the separator are more stringent due to the volume changes and sharp dendrite formation during Zn deposition/stripping at the anode [156]. The separator direct contacts with the anode and cathode, so it is expected to regulate the chemistry of the electrode/electrolyte interface through modification of the separator [157]. Glass fibers (GFs) are commonly used as separators in AZMBs, which possess fast ionic conductivity but poor mechanical strength. Moreover, the uneven porous structure of GFs allows Zn dendrites to grow wantonly [158]. Therefore, the ideal separator should feature sufficient porosity, mechanical flexibility, ionic conductivity, ion-selective channels, and excellent electrolyte absorption and retention capacities [159, 160]. At present, one of the main strategies used to control the separator is to modify traditional GFs, e.g., by introducing zincophilic coating to increase the Zn^2+^ ion concentration at the anode surface and reduce the nucleation overpotential. Another widely adopted strategy is to develop a new membrane exhibiting high zincophilicity, mechanical flexibility, and electrolyte wettability [161]. These modification strategies are effective in promoting uniform Zn deposition at high Zn utilization and restraining side reactions. Table 3 provides a comprehensive summary.

**Table 3:** Summary of electrochemical performance of AZMBs with Zn foil anodes for separator designing in terms of different parameters

Anode/thickness of Zn foil (μm)^a^	Separator	Electrolyte^b^	Cathode	Symmetric cells		Full cells		Refs.
				Cycling performance (mAh cm^−2^ /lifespan/mA cm^−2^)	Zn utilization (%)^c^	Cycling performance (mAh g^–1^/cycles/A g^–1^ /capacity retention)	N/P	
Zn (100 μm)	NG/GF	2 M ZnSO_4_	MnO_2_	25/48 h/20	42.7	198.6/1000/5/79%	–	[70]
Zn (100 μm)	VVLP	2 M ZnSO_4_	MnO_2_	10/350 h/10	17.08	–/70/0.1 C/79.1%	1.35	[79]
Zn (10 μm)	CNF-SO_3_Zn	–	PANI	5/60 h/2	80	100/150/0.2/95%	–	[155]
Zn (10 μm)	ZnHAP/BC	2 M ZnSO_4_	V_2_O_5_	–/611 h/–	80	201.4/2500/10/82%	2.7	[156]
Zn (50 μm)	CF	2 M ZnSO_4_	(LPC)/α-MnO_2_	20/300 h/2	69.7	175.6/1000/1/87.7%		[158]
Zn (13 μm)	BC hydrogel electrolyte	2 M ZnSO_4_	NVO	6.5/100 h/6.5	85	212/1000/5/87.7%		[159]
Zn (13 μm)	BC	2 M ZnSO_4_	NVO	6.5/100 h/6.5	85	212/1000/5/83%	–	[161]
Zn (85 μm)	Janus	3 M ZnSO_4_	MnO_2_	28.3/220 h/28.3	56	–/1900/1/95%	–	[162]

^a^Thickness of Zn foil (μm) for symmetric cells

^b^Electrolyte of symmetric cells

^c^The Zn utilization is calculated using Eq. (2)

**Fig. 5:**
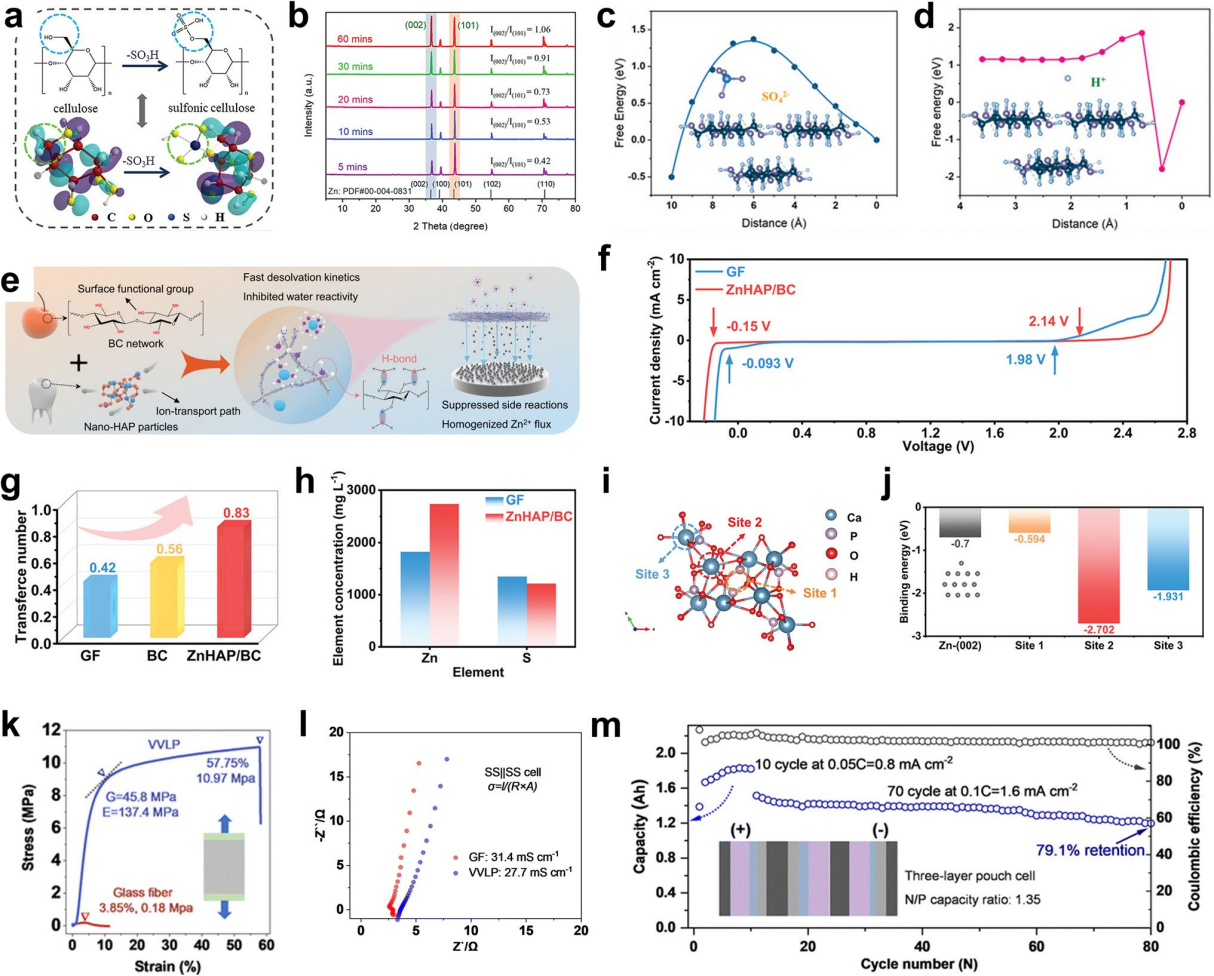
**a** Synthesis of sulfonic cellulose by grafting sulfonic acid groups on the cellulose backbone. **b** XRD patterns of Zn anodes after deposition by employing the Janus separator. Energy profiles of **c** SO_4_^2−^ and **d** H^+^ adsorption on the sulfonic cellulose under zeta potential. Adapted from Ref. [162]. Copyright 2022, John Wiley and Sons. **e** Schematic illustration of the nature-inspired ZnHAP/BC design and its effect in regulating Zn deposition behavior. **f** Electrochemical stability window (ESW) of the electrolyte in different battery systems. **g** Comparison of the Zn^2+^ transference number of different separators. **h** Zn^2+^ permeability of the GF and ZnHAP/BC separator. **i** Calculation model for the interaction of Zn^2+^ and HAP with possible adsorption sites and **j** the corresponding binding energies. Adapted from Ref. [156]. Copyright 2023, John Wiley and Sons. **k** Loading curves under uniaxial elongation and **l** impedance profiles for ionic conductivity measurements of different separators. **m** Cycling performance and areal capacity of pouch cell at a rate of 0.05 C (10 cycles) and 0.1 C (70 cycles) with N/P of 1.35. Adapted from Ref. [163]. Copyright 2023, Royal Society of Chemistry

Janus membrane-absorbed sulfonated cellulose graphene was employed to modify a commercial GF separator [162]. The hydrophilic sulfonated cellulose improved the wettability of the separator and electrolyte, and the sulfonic groups on the cellulose facilitated the adsorption of Zn^2+^ ions (Fig. 5a). The oriented graphene adsorbed on the diaphragm provided the preferred (002) texture for Zn deposition (Fig. 5b). With further deposition, the Janus separator continuously adjusted the growth morphology of Zn on the exposed Zn (002) surface and promoted the epitaxial growth of Zn. In addition to regulating the Zn deposition morphology, the ion-sieving Janus separator provided a single Zn^2+^ ion channel and enriched the Zn^2+^ ions on the anode surface. Because the sulfate radical reached the high energy barrier of the sulfonated cellulose surface and the hydrogen ions formed hydrogen bonds with the hydroxyl groups on the cellulose skeleton, it was anchored on the sulfonated cellulose surface with the lowest energy (Fig. 5c, d), thus reducing the occurrence of side reactions. The Zn symmetric batteries with Janus separators maintained stable cycling for 220 h at 28.3 mA cm^−2^/28.3 mAh cm^−2^, with a corresponding DOD value of more than 56%.

There are abundant sources of biomass cellulose (BC) in nature. The films prepared by biomass cellulose possess good hydrophilicity, abundant hydroxyl groups, strong mechanical properties, and a uniform porous structure. Therefore, BC is an ideal material for constructing the separator. The BC is rich in functional groups (–OH) (Fig. 5e) [156]. A ZnHAP/BC membrane was prepared by hydrogen bonding self-assembly method and modified with nanohydroxyapatite (HAP). The wide electrochemical window showed that the hydroxyl ZnHAP/BC separator significantly reduced the reactivity of water (Fig. 5f). Additionally, Zn was enriched on the anode surface due to the blocking effect of the negative oxygen functional groups on the SO_4_^2−^ in ZnHAP/BC, indicating that ZnHAP/BC enhanced the Zn^2+^ ionic conductivity by accelerating desolvation and migration of the Zn^2+^ ions (Fig. 5g, h). In addition, considerable zincophilic adsorption sites regulated the uniform Zn^2+^ flux toward the anode/electrolyte interface, thus promoting uniform Zn deposition (Fig. 5i, j). Polymers are also effective separator materials. Zhou et al. used a robust hydrophilic polyvinylidene difluoride-type filter as a separator (VVLP) [163]. Compared with GFs, the VVLP showed higher mechanical strength (Fig. 5k) while maintaining an ionic conductivity similar to that of the GFs (Fig. 5l). In addition, the uniformly distributed pore sizes (500 nm) provided uniform channels for ion transport, which led to a more uniform Zn^2+^ ion concentration gradient and fine Zn dendrite particles with adjustable size. Moreover, with high capacity, the coverage of the hydrated zinc sulfate (ZSH, (Zn(OH)_2_)_3_ZnSO_4_·*x*H_2_O) composite layer effectively reduced the porosity of the VVLP separator, triggering the mechanical inhibition that tended to flatten the interface of the electrodeposition zone. When the N/P was 1.35, a high capacity of 1.83 Ah was obtained with a three-layer high-energy pouch cell. At a discharge voltage of 1.35 V, the energy density reached 115.1 Wh kg^−1^ (Fig. 5m).

Designing novel separators is an effective strategy to improve Zn utilization by promoting homogeneous Zn^2+^ ion flux and inhibiting dendrite growth. Utilizing functionalized separators can reduce costs and increase energy density by avoiding the use of expensive and thick glass fibers. However, separator designing strategies to improve Zn utilization have received less attention, and existing strategies are limited in improving Zn utilization. Solving both cathode side and anode side problems is pivotal to achieve highly stable AZMBs with high Zn utilization. It is necessary to further develop functionalized separators with simple preparation methods and good mechanical properties. Additionally, strategies such as electrolyte modification and interfacial protective layers should be employed synergistically with the separator designing to achieve high Zn utilization for AZMBs.

## Pre-deposited Zn Metal Anodes

It is easy to reduce the amount of excess Zn by reducing the thickness of the Zn foil. However, if the amount of Zn is excessively reduced, the overly thin Zn foil (< 10 μm) will be quickly destroyed during cycling and fail as a substrate for depositing Zn [164]. Therefore, to build stable Zn anode structures while reducing the amount of excess Zn and increasing Zn utilization, constructing suitable collectors for deposited Zn is an effective strategy [165]. Designs of 3D or gradient anodes can increase the contact area between the electrode and the electrolyte and the number of Zn nucleation sites while reducing the local current and nucleation overpotential, resulting in a uniform electric field and distribution of Zn^2+^ ions to enable uniform Zn deposition and slow the dendrite growth [46, 166]. Moreover, the 3D skeleton with good mechanical strength and toughness can adapt well to the volume changes of the anode during cycling [167, 168]. In addition, the dendrites can be better inhibited by low lattice mismatch of the collector/Zn interface [169–171]. The materials used in the skeleton of collector can be divided into carbon materials [98, 172–179], Zn [170, 180], other metals such as Cu [164, 167, 171, 181, 182], Ni [183], Ti [184], Al [185], and Ag [186], alloy materials [187–190], MXenes [191, 192], and MOF-based materials [193]. In brief, the preparation of Zn metal anodes by pre-deposition can reduce the amount of excess Zn and thus improve the Zn utilization. Table 4 provides a comprehensive summary.

**Table 4:** Summary of electrochemical performance of AZMBs with pre-deposited Zn metal anodes in terms of different parameters

Anode/capacity of pre-deposited Zn (mAh cm^−2^)	Electrolyte^a^	Cathode	Symmetric cells		Full cells		Refs.
			Cycling performance (mAh cm^−2^/lifespans/mA cm^−2^)	Zn utilization^b^	Cycling performance (mAh g^−1^/cycles/A g^−1^/capacity retention)	N/P	
*Carbon-based*							
ZnFe-PBA@CC (–)	2 M ZnSO_4_	PVO	0.5/1320 h/5 (Asymmetric cells)	99.48% (CE)	214 Wh kg^−1^ /360/3/62%	1.2	[46]
Epitaxial Zn	2 M ZnSO_4_	α-MnO_2_	3.2/2500/40 (Asymmetric cells)	99.7% (CE)	–/1000/8 mA cm^−2^/–	2	[98]
Zn@CNF (5)	2 M ZnSO_4_	V_2_O_5_	2/488 cycles/0.5	40%	133.4/260/0.5/92.1%	2.4	[165]
3D-RFGC@Zn (1–120)	2 M ZnSO_4_	V_2_O_5_@3D-LC	80/2600 cycles/80	–	156.2/2400/40.0 mA cm^−2^_/_–	–	[166]
Cu NBs@NCFs-Zn (8)	2 M ZnSO_4_	Mn_2_O_3_-ZnMn_2_O_4_	2/250 h/5	25%	130.4/2000/1/67.6%	–	[168]
Zn/CNT (6.67)	2 M ZnSO_4_	MnO_2_	2.5/110 h/5	35%	167/1000/20 mA cm^−2^/88.7%	–	[172]
3DGs@Zn (10)	2 M ZnSO_4_	V_2_O_5_	1/1100 h/2	10%	431.5/150/4 mA cm^–2^	1.74	[173]
Zn@CFs (7.85)	2 M ZnSO_4_	a-MnO_2_	1/150 h/1	12.7%	239.4/140/1 C/86.8%	–	[174]
Zn/CNT (1.8–5.0 mg cm^−2^)	2 M ZnSO_4_	a-MnO_2_/CNT	667 mA h g^–1^/2000 min/4 A g^–1^	81%	172/1000/10 C/–	–	[175]
Cu/*C*_flower_/Zn (1, 2, 10 mAh)	3 M ZnSO_4_	MnO_2_	1/1000 h/2	–	275/100/0.1	41.3% (DOD for full cells)	[176]
Zn@ZnO&CFs (10 mAh cm^–2^)	ZnSO_4_	MnO_2_	5/150 h/10	50%	122.24/1500/1/87%	–	[177]
Zn@PTCC (3.33)	2 M ZnSO_4_	VOH	2/480 h/2	60%	205.3/500/1/95.0%	2.86	[178]
Zn@NCC (10)	3 M Zn(CF_3_SO_3_)_2_		0.5/700 h/1	10%	–	–	[179]
*Metal-based*							
(C_2_F_4_)_*n*_–C@Cu@Zn (5)	3 M Zn(CF_3_SO_3_)_2_	V_2_O_5_	2/900/5	40%	140/2500/3/88.35%	3.3	[164]
SF-ly@Cu–Zn (12 mg cm^−2^)	2 M ZnSO_4_	MnO_2_	10/1100/10	83%	151/2000/1.5/88.8%	–	[167]
Zn@CuNWs (20)	2 M ZnSO_4_	Mn_0.25_V_2_O_5_	5/180 h/20	25%	281.9/800/5/91.4%	–	[169]
zinc-plated P-Cu (–)	2 M ZnSO_4_	MnO_2_	2/1100 cycles/5 (Asymmetrical cell)	99.77% (CE)	–/640/5 mA cm^–2^/93.9%	20% (Zn utilization for full cells)	[171]
Zn film (4.9 mg cm^−2^)	3.0 M Zn(CF_3_SO_3_)_2_	PVO	2/250/5	–	283.3/1000/10/87.6%	–	[180]
Cu mesh (5)	1 M ZnSO_4_ + 0.5 M Na_2_SO_4_ + 1 g L^−1^ PAM	MnO_2_	4/280 h/2	80%	–/600/1/98.5%	–	[181]
Zn@Bio-scaffold (6)	2 M ZnSO_4_	VOH	1/4000 cycles/5 (Asymmetrical cell)	99.83% (CE)	295/500/2/48.2%	–	[182]
3D Ni–Zn (8)	2 M ZnSO_4_	PVO	5/300/2	62.5%	314/1000/10/80%	–	[183]
Zn@3D Ti-TiO_2_ (5)	2 M ZnSO_4_	S-MXene@MnO_2_	1/2000/1	20%	226.7/100/0.5/98.49%	–	[184]
Al@C foil (–)	1 M Zn(OTf)_2_ γ-valerolactone	AC	1/40 h/1 (Asymmetrical cell)	99.48% (CE)	–/2000/5 mA cm^–2^/–	1.5	[185]
Zn@a-Ag mesh (2)	2 M ZnSO_4_	LFP	0.5/7000/5 (Asymmetric cell)	–	88/100/0.5 mA cm^–2^_/_	4	[186]
ZnSn-1 (–)	2 M ZnSO_4_	V_2_O_5_	5/240/5	35.2%	209.2/113/5/–	–	[187]
DES-Zn (–)	2 M ZnSO_4_	AC	2/300/3	40%	–/7000/4/70.9%	25% (DOD for full cells)	[188]
Zn@ LM-AgT (10)	2 M ZnSO_4_	NVO	2/200 h/2	20%	163.7/1000/2/–	–	[189]
InZn (–)	2 M ZnSO_4_	DTT	5/1000 h/1	45%	110/40/0.1/–	–	[190]
Zn@TG (10)	2 M ZnSO_4_	MnO_2_@N–C@CC	2.5/400/5	25%	208/330/2/79%	–	[194]
*Others*							
MGA@Zn (5)	2 M ZnSO_4_	LMO	1/1100/10	20%	60/480/2 C/–	–	[76]
ZnGaIn||Mxene (–)	2 M ZnSO_4_	α-MnO_2_	1/600/1	–	150/400/3.2/–	–	[192]
Zn@ZIF-8–500 (10)	2 M ZnSO_4_	I_2_	1/50/1	10%	–/1000/2/97%	–	[193]

^a^Electrolyte of symmetric cells

^b^The Zn utilization is calculated using Eq. (3)

**Fig. 6:**
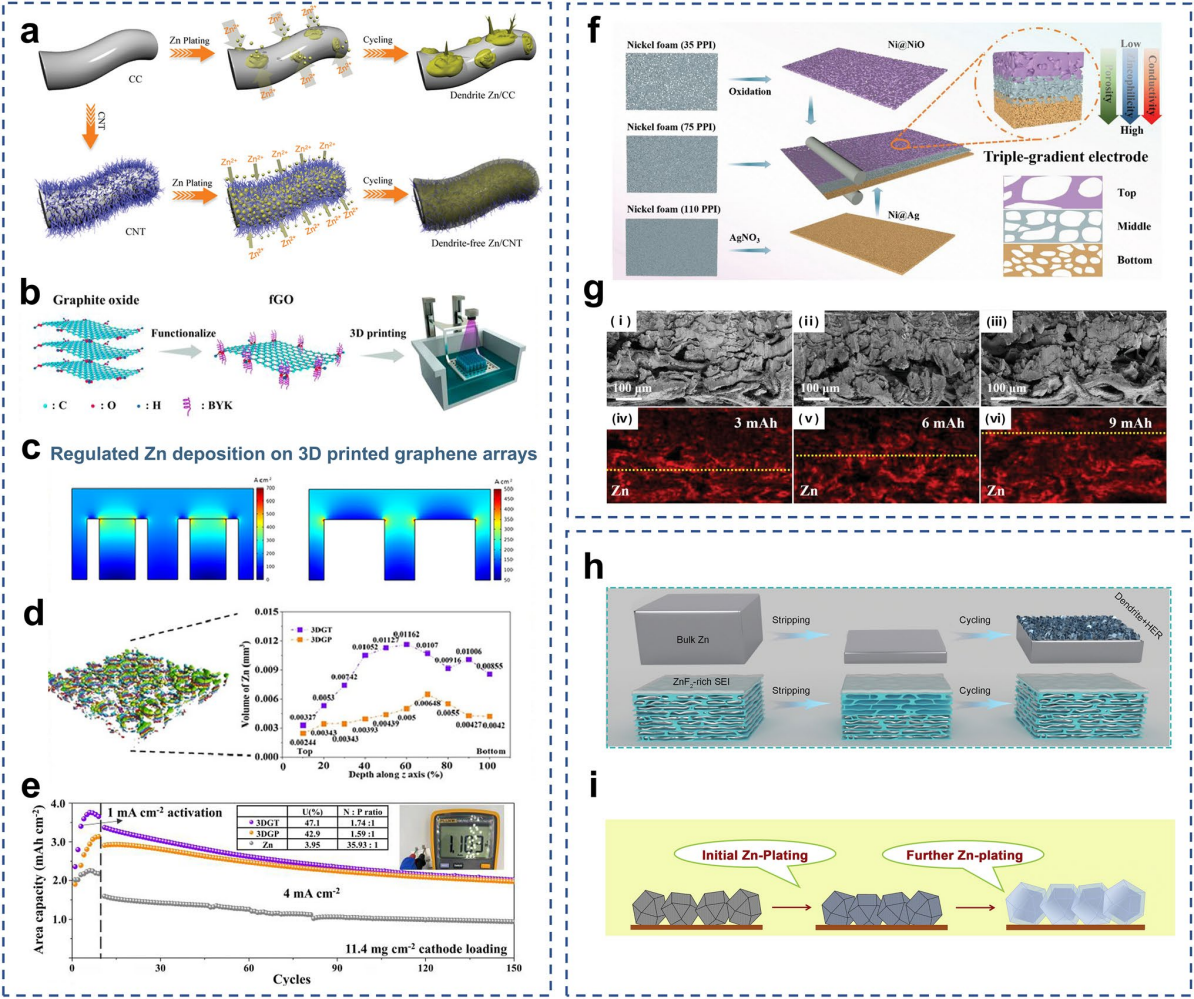
**a** Schematic illustrations of Zn deposition on CC and CNT electrodes. Adapted from Ref. [172]. Copyright 2019, John Wiley and Sons. **b** The digital light processing (DLP) fabrication of the 3D-printed graphene arrays. **c** Multi-physics models of the current density distributions predicted for the 3DGT and 3DGP. **d** The Micro-CT images of 3DGP and the electrodeposited Zn volume of 3DGT and 3DGP. **e** Cycling performance of 3DGT@Zn||V_2_O_5_, 3DGP@Zn||V_2_O_5_, and Zn||V_2_O_5_ in pouch cell at 4 mA cm^−2^, their N/P and the Zn utilization (U) are presented in the inset table. Adapted from Ref. [173]. Copyright 2023, Elsevier. **f** Schematic illustrations of preparation and characterization of the triple-gradient electrode. **g** Cross-sectional SEM images and corresponding Zn element mapping of the 35Ni@PVDF/75Ni/110Cu triple-gradient electrode after Zn deposited at 5 mA cm^−2^ for different capacities. Adapted from Ref. [194]. Copyright 2022, John Wiley and Sons. **h** Schematic illustration of Zn plating and cycling on bulk Zn and MGA@Zn electrodes. Adapted from Ref. [191]. Copyright 2021, John Wiley and Sons. **i** Schematic diagram of Zn electroplating/stripping on ZIF-8-500 electrode. Adapted from Ref. [193]. Copyright 2019, Elsevier

As mentioned above, carbon materials can be used as surface coatings to protect Zn anodes [76–79, 97]. 3D structured carbon materials such as carbon fibers and carbon nanotubes possess excellent electrical conductivities, light weights, high porosities, and good mechanical strengths [174, 175]. Therefore, they can also be used as the host materials for pre-deposition. The carbon nanotube networks are highly conductive backbones for Zn deposition, and they have low Zn nucleation overpotentials (Fig. 6a) [172]. The uniformly distributed electric field can promote the dendrite-free Zn deposition. This pre-deposited host structure achieved a DOD of 28%. 3D printing is an advanced technique used in building 3D structures, which allows precise control of the size and distribution of the pore structure and directional modulation of the charge transport paths [183]. By combining graphene materials with 3D printing technology, Zeng et al. fabricated 3D-printed graphene (3DG) arrays in the form of tubes and pillars (Fig. 6b) [173]. 3DGT/P altered the distribution of Zn^2+^ ions by modulating the electric field so that Zn was preferentially deposited in the 3DG, avoiding large stress on the membrane (Fig. 6c). The volume distribution of Zn deposited along the z-axis can be seen in Fig. 6d. The volumes of deposited Zn metal in the top layers of the 3DGT and 3DGP were much lower than those in the deeper layers. 3DGT@Zn||V_2_O_5_ and 3DGP@Zn||V_2_O_5_ pouch cells showed high Zn utilization (47.12% and 42.94%) and areal energy densities of 3.27 and 2.72 mWh cm^−2^ (Fig. 6e).

In addition to carbon, metals are also introduced to design 3D structures. Compared to 2D planar Zn foils, microporous engineered Zn micromesh has excellent flexibility and mechanical strength and offers better electrolyte wetting [180]. The Zn micromesh can provide an ordered distribution of ion concentration and electric field, allowing Zn^2+^ ions to preferentially nucleate and grow on the inner walls of the micropores while enabling dendrite-free deposition of Zn. However, 3D Zn structures are susceptible to side reactions such as corrosion and structural damage during repeated cycling at high Zn utilization. Guan et al. prepared a triple-gradient electrode with electrical conductivity, zincophilicity, and porosity generated by simple mechanical roller pressing [194]. Nickel foams with different porosities were used as the substrates. The Ag in the bottom layer provided good electrical conductivity and zincophilicity, the NiO in the top layer was a semiconductor with poor zincophilicity, and the Ni in the middle layer had moderate electrical conductivity and zincophilicity (Fig. 6f). The triple-gradient electrode allowed regulation of the electric field, the Zn^2+^ ion flux, and the Zn deposition paths in Zn anodes, enabling bottom-up deposition of Zn metal and preventing short circuits due to overgrowth of dendrites piercing the septum (Fig. 6g).

Other materials such as MXenes and MOFs can also be utilized as host materials for depositing Zn. Chen et al. prepared a hybrid aerogel (MGA) rich in zincophilic sites and porous channels by assembling MXene with graphene (Fig. 6h) [191]. The fluorine-containing functional groups of MXene form ZnF_2_ with the initially deposited Zn as the SEI to inhibit the formation of dendrites. The mechanical toughness of the MGA can be adjusted to the volume changes due by controlling the thickness during the deposition process. Therefore, encapsulation of the deposited Zn by the MGA was achieved. A capacity retention of 91% was realized at N/P ≈ 1.67. The ZIF-8 annealed at 500 °C (ZIF-8-500) (Fig. 6i) had a trace Zn metal, porous organic ligands, and highly ordered hierarchical porous structures [193]. The trace amount of Zn^0^ in the framework of ZIF-8-500 showed excellent performance that CE was close to 100% and a dendrite-free characteristic. The large number of zincophilic sites in the ZIF-8-500 with the graded porous structure effectively guided the Zn deposition, and the residual Zn^0^ was used as a backup Zn source. The high reversibility and excellent full cell lifespan at high energy density were achieved. The pre-plated Zn (Zn@ZIF-8-500) was coupled with the I_2_ cathode to form the I_2_||Zn battery, showing a super long life of 1600 cycles and a high energy density of 140.8 Wh kg^−1^.

Although pre-deposited Zn anodes can approach high Zn utilization even at low capacity, such anodes with low Zn capacity still possess some limitations in practical applications compared to Zn foil anodes. The large mass and volume of the pre-deposited substrate reduce the energy density of full cells. In addition, when 3D structures are used as hosts for Zn deposition, the top side often lacks effective protection. As deposition progresses, the side reactions such as HER will be intense. Therefore, for pre-deposited 3D collector structures, lighter materials are required to accommodate increased deposition and discharge capacity. It is also necessary to synergistically optimize 3D structures and surface protection layers for stable and reversible Zn metal anodes.

**Fig. 7:**
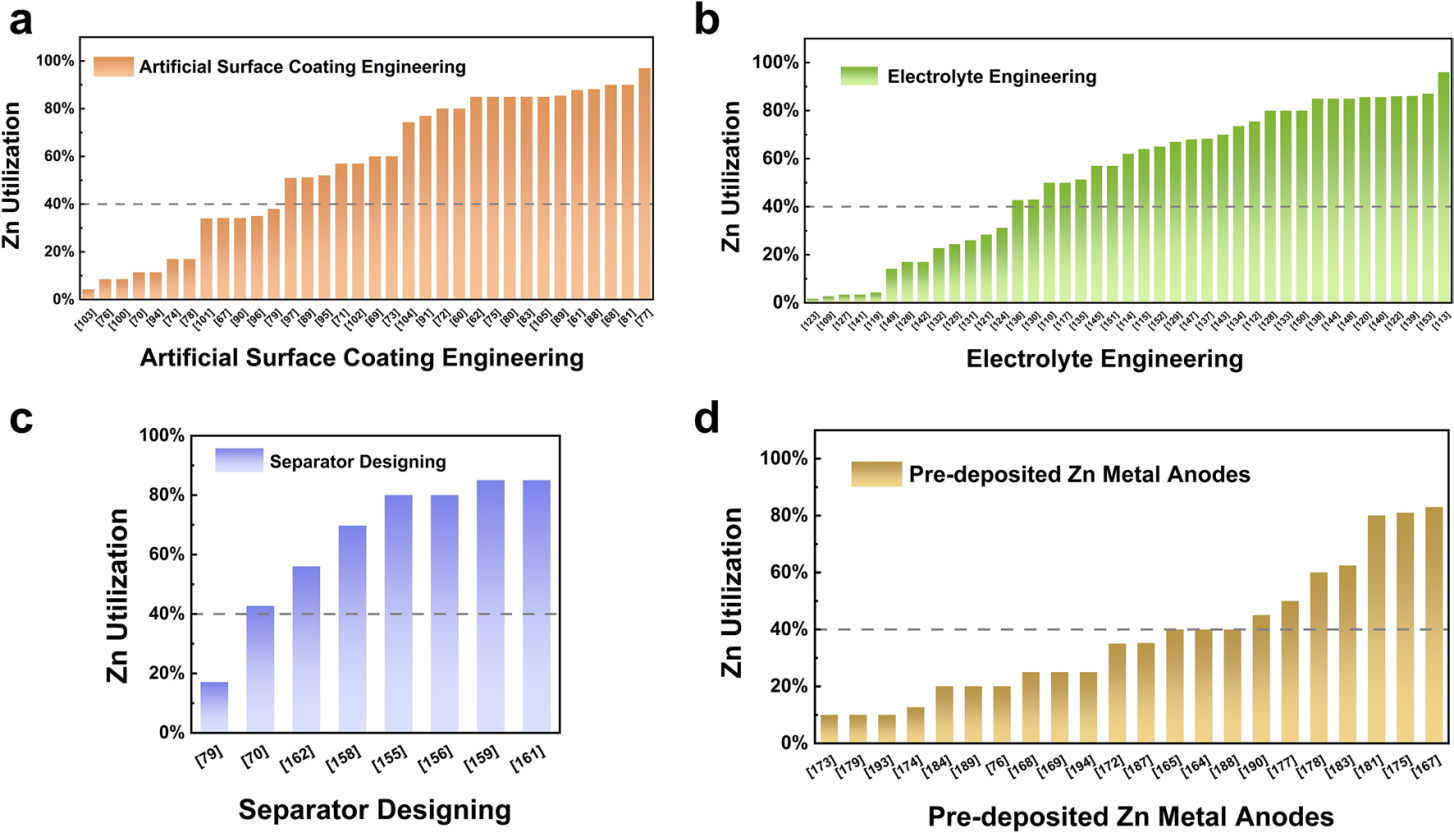
Comparison of Zn utilization of AZMBs using different modification strategies

In previous studies, achieving a DOD of 40% was identified as a reasonable target to evaluate AZMBs for practical applications [165]. The Zn utilization using different modification strategies is summarized in Fig. 7, with a value of 40% as the evaluation criterion. The Zn utilization of AZMBs can be improved by using different Zn anodes to reduce the Zn amount. In addition, the Zn utilization is also affected by other factors, such as the areal capacity during charge/discharge, which is reflected in Eq. 1. Therefore, increasing the areal capacity while reducing the Zn amount is an effective strategy to further improve the Zn utilization of AZMBs.

## Anode-Free Aqueous Zn Metal Batteries

**Fig. 8:**
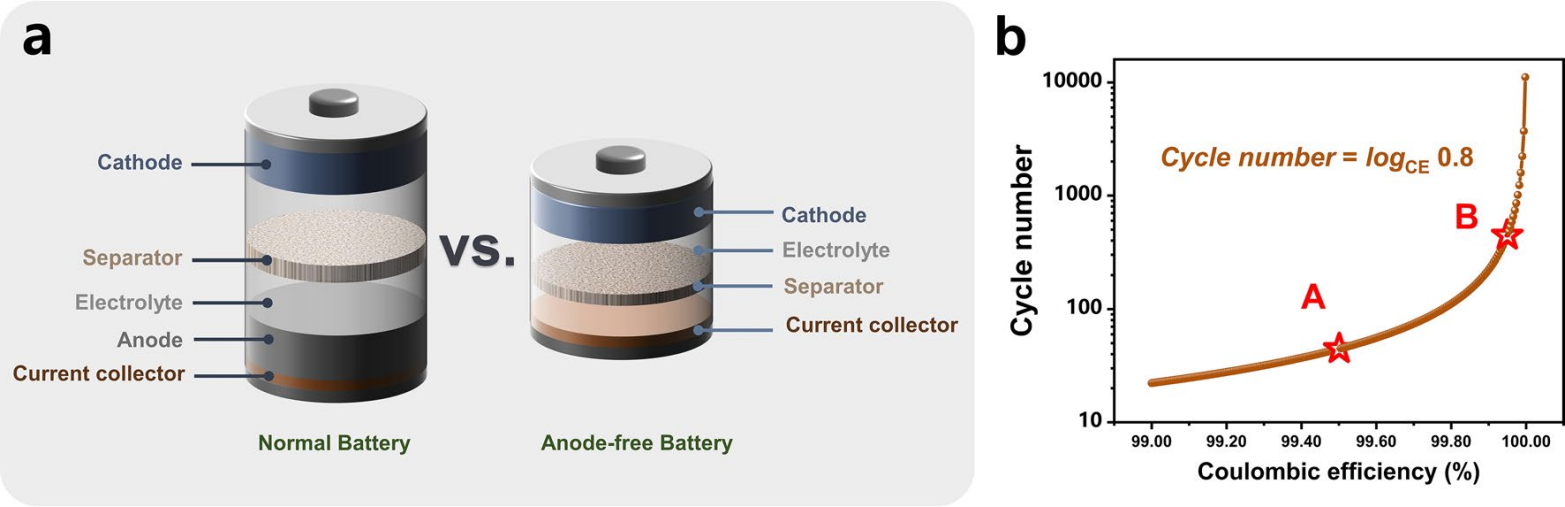
**a** Schematic illustrations of a normal battery with a Zn metal anode and an anode-free battery. **b** Plot of the calculated cycle number of a battery by the CE of the anode

Anode-free batteries have recently been proposed and widely studied in lithium and sodium metal batteries [47]. Benefiting from the excellent theoretical capacity of Zn, the anode-free configuration was introduced into AZMBs and further optimized [54]. Unlike AZMBs using Zn foils or pre-deposited Zn as anodes, the AF-AZMBs are characterized by the light weight attributed to eliminating the excess Zn (Fig. 8a). Almost all Zn sources in the AF-AZMBs are utilized and contribute to the battery capacity. This distinctive design maximizes the energy density beyond those of traditional metal-based batteries. The configuration of AF-AZMBs differs significantly from normal AZMBs, with a Zn-free current collector as the anode and a pre-intercalated cathode or Zn-salt electrolyte as the only Zn reservoir. When AF-AZMBs are assembled, the batteries are fully discharged and in a low-energy state. After the first full charge, Zn^2+^ ions pre-embedded in the cathode or electrolyte are removed and deposited on the anode. Therefore, the DOD of AF-AZMBs can approach 100%. Nevertheless, the AF-AZMBs generally show poor stability. A critical factor for stable AF-AZMBs is the CE of Zn plating/stripping, which determines the battery reversibility and actual cycling lifespan. When the Zn source in the battery becomes limited, any slight loss of Zn will significantly reduce the cycling lifespan. The formula used to calculate the cycle number for different CE is as follows:

9 $${\text{CE}}^{{\text{Cycle number}}} = 80\%$$

10 $${\text{Cycle number}} = \log_{{{\text{CE}}}} 0.8$$

Theoretically, according to Eq. (10), if the battery is defined to fail with 80% capacity retention, 99.5% of the CE sustains only 45 cycles (Fig. 8b). When the CE achieves 99.95%, the lifespan of the battery will be approximately 450 cycles. In AF-AZMBs, the dendrites are directly formed on the collector surface, which is a crucial parameter affecting the CE [195]. Excess growth of dendrites to form “dead Zn” and side reactions that generate byproducts cause irreversible wastes of Zn and decrease the CE [196, 197]. Therefore, constructing dendrite-free Zn anodes and inhibiting interfacial side reactions are required to improve the electrochemical performance of AF-AZMBs. Recent strategies used in constructing AF-AZMBs will be described in terms of anode current collector engineering and electrolyte engineering. Table 5 provides a comprehensive summary.

**Table 5:** Summary of electrochemical performance of AF-AZMBs in terms of different parameters

Anode	Cathode	Electrolyte^*a*^	Asymmetric cells		Anode-free cells		Refs.
			Cycling performance (mAh cm^−2^/cycles/mA cm^−2^)	CE	Cycling performance (mAh g^−1^/cycles/A g^−1^/capacity retention)	Energy density (Wh kg^−1^)	
C/Cu	β-MnO_2_	3 M Zn(CF_3_SO_3_)_2_ + 0.1 M Mn(CF_3_SO_3_)_2_	0.5/300/1	99.6%	100%/300	135	[54]
Cu-Ag	graphite	3 M Zn(TFSI)_2_/EMC	0.5/200/0.5	99.86%	84/1000/0.5/82%	79	[55]
CuNC@Cu	G/PVP@ZnI_2_	5 mM ZnI_2_ + 10 mM I_2_ + 2 M ZnSO_4_	1/1700 h/5	99.88%	–/1000/1/80%	162	[196]
AgNWA@Cu	ZnMO	2 M ZnSO_4_	10/200/40	99.8%	230/600/0.5/73%	–	[197]
Ti_3_C_2_T_*x*_/Nanocellulose hybrid film (18.3 μm)	LMO	2 M Li_2_SO_4_ + 1 M ZnSO_4_ + 0.08 M ZnF_2_	2/1000/5	–	42.4/2000/1/81.5%	–	[199]
Ti_3_C_2_T_*x*_/Nanocellulose hybrid film (6.2 μm)	NaVPO	18 M NaClO_4_ + 2 M Zn(CLO_4_)_2_	2/300/5	98.1%	71.3/1000/2/84.6%	–	[200]
Cu@AOF	Zn_0.5_VO_2_	2 M ZnSO_4_	1/6000/10	99.90%	203/2000/1/60%	–	[201]
ZnA@Cu	α-MnO_2_	2 M ZnSO_4_ + 0.2 M MnSO_4_	1/6700/20 (symmetric cells)	–	–/907500/2/74.6%	192.8	[202]
Sb/Sb_2_Zn_3_-HI@Cu	carbon felt	0.5 M ZnBr_2_ + 0.25 M TPABr	10/550 h/20	98%	10 mAh cm^−2^/800/10 mA cm^−2^/–	274	[203]
Cu foil	LFP	4 M ZnSO_4_ + 2 M Li_2_SO_4_ + 10 Vol% DME + 0.005 M SnBr_2_	1/140 h/0.5	–	126/100/0.5 C/35.2%	–	[204]
Cu@Cu_3_Zn-modified CCF	Zn_3_V_3_O_8_	3 M Zn(CF_3_SO_3_)_2_	0.5/300/1	99.2%	–/200/2/80%	–	[205]
ZA@3D-nano Cu	MnO_2_	1 M ZnSO_4_ and 1 M MnSO_4_	2/1100 h/4	98.4%	450 mAh g^–1^_MnO2_/1000/10 mA cm^–2^/89%	–	[206]
MDC–Cu	Zn/Mn-MOF@CNT	2 M ZnSO_4_	0.5/3000/1	99.4%	–/900/5 C/92%	1.34 (N/P)	[207]
ZCDC-Cu	Zn-Mn MOF@CNT	2 M ZnSO_4_	0.5/4250/1	99.6%	160.3/5000/3 C/–	1.23 (N/P)	[209]
*Electrolyte*							
Stainless steel	LiMn_2_O_4_	0.08 M ZnF_2_ + 2 M ZnSO_4_ + 1 M Li_2_SO_4_	3/1000/40	99.14%	59/100/0.2/75.64%	–	[210]
gr-Cu	Zn_*x*_CVO	3.4 M ZnSO_4_ in water + 0.5M Zn(TFSI)_2_ in AN	0.1/300/0.1	99.6%	141/100/1/76.7%	183	[212]
Cu	Zn_0.25+*x*_VO	2 M Zn(OTf)_2_ in 7:3 sulfolane/water	4/200/2	99.9%	4 mAh cm^−2^/100/0.15 C/85%	–	[213]
Cu foil	LiFePO_4_	2 M Zn (OTf)_2_ + 1 M LiOTf + 70 vol% EG/H_2_O	0.5/1000 h/2	99.84%	–/100/1 mA cm^−2^ /75.2%	–	[214]
Ti	Zn_*x*_VOPO_4_	4 M Zn(OTF)_2_ + 0.5 M Me_3_EtNOTF	0.5/1000/0.5	99.9%	–/90/0.5 mA cm^−2^/80%	–	[215]
Cu foils	Carbon felt	1 M ZnSO_4_ + 1 M LiCl + 0.4 M TMACl	5/80/10	99.6%	0.95 mAh/200/20 mA cm^−2^/–	–	[216]
Cu	Z-PANI	ASE	1/90/1	99.9%	121.7/300/1/78.8%	–	[217]
Cu	ZnMn_2_O_4_	50% PC-sat	0.5/500/1	99.93%	350 mA g^−1^/275/0.5 mA cm^−2^/80%	–	[218]

^a^Electrolyte of anode-free cells

### Anode Current Collector Engineering

Zincophilic materials (e.g., Cu foil) are usually used as the current collectors for the anodes in AZMBs due to their low activities for the competitive HER [198]. Nevertheless, if these current collectors are directly utilized, the low CE and uncontrollable dendrite formation will seriously affect the performance of AF-AZMBs. Therefore, some strategies for designing and modifying the current collectors have been proposed to improve the performance of AF-AZMBs, such as designing surface protection layers on the current collectors [54, 55, 199–202], alloying the anode surfaces [203, 204], and constructing 3D nanostructure hosts [196, 197, 205–207].

#### Anode Surface Coating Engineering

**Fig. 9:**
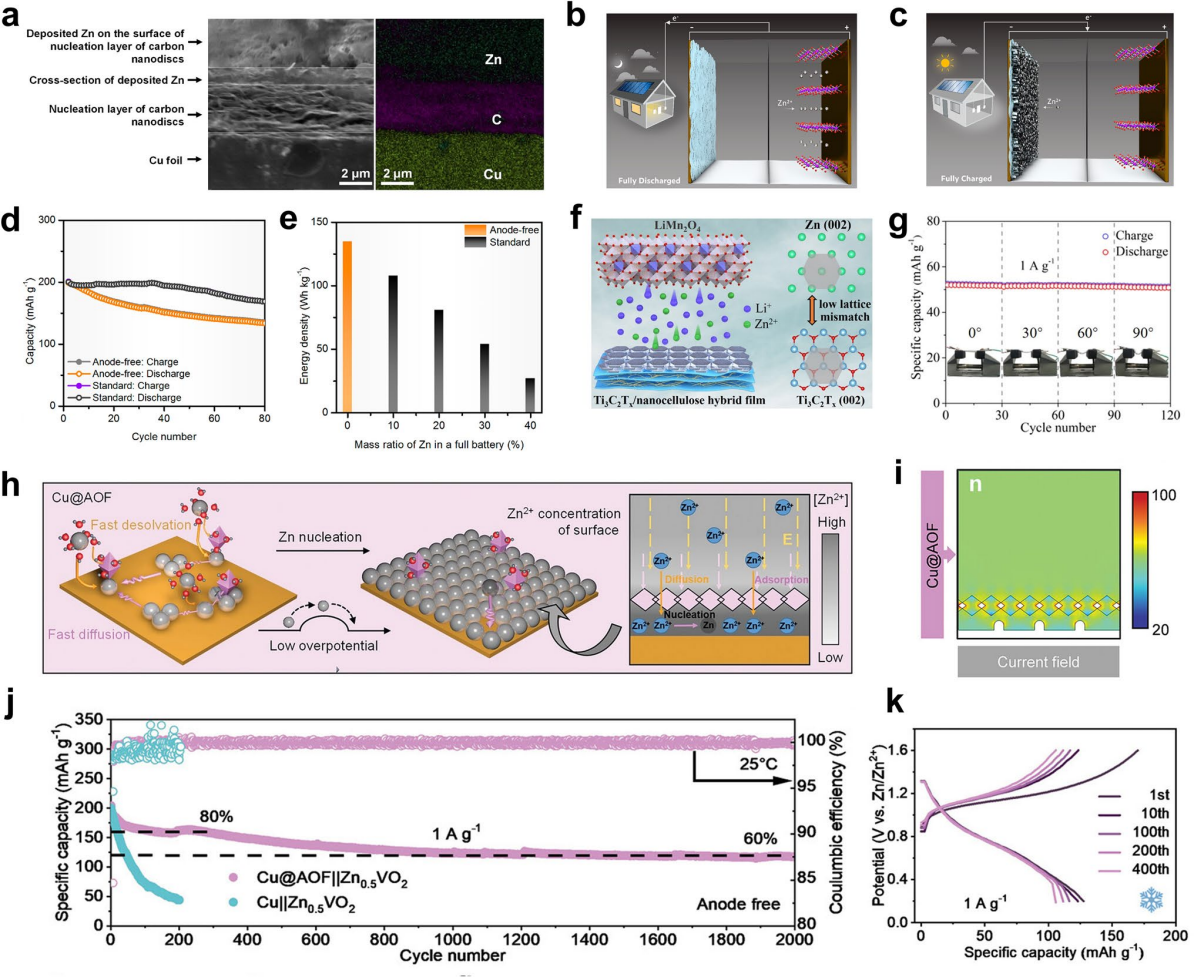
**a** Cross-sectional SEM image and the corresponding EDS mapping of the C/Cu electrode after plating Zn. Schematic demonstrations of the anode-free Zn||MnO_2_ battery in** b** fully discharged and **c** charged states. **d** Cycling performance of standard and anode-free Zn||MnO_2_ batteries. **e** Comparison of the energy densities of the anode-free and standard batteries. Adapted from Ref. [54]. Copyright 2021, American Chemical Society. **f** Schematic illustration of the quasi-solid-state hybrid Zn-Li battery. **g** Bending affordability test of the quasi-solid-state hybrid Zn-Li battery. Adapted from Ref. [199]. Copyright 2022, American Chemical Society. **h** Schematic diagram of the Zn deposition on Cu@AOF surface. **i** 2D phase-field simulation of current density and Zn^2+^ concentration profiles of Cu@AOF. **j** Cycling performance of Cu||Zn_0.5_VO_2_ full cells using bare Cu and Cu@AOF at a current density of 1 A g^−1^. **k** Charge/discharge voltage curves of Cu||Zn_0.5_VO_2_ full cells using Cu@AOF after different cycles at a low temperature of − 20 °C. Adapted from Ref. [201]. Copyright 2023, John Wiley and Sons

Surface modification has been utilized to avoid Zn dendrite formation and side reactions at anode/electrolyte interfaces in AZMBs. When applied to AF-AZMBs, the coating layers should ensure high Zn^2+^ conductivity and robust adhesion to the anode surface. However, a large volume and mass of the coating layer will decrease the energy density. Cui et al. demonstrated a first workable anode-free Zn-MnO_2_ battery [54]. The anode was a Cu foil coated with a nucleation layer of carbon nanodiscs (C/Cu anode). The pre-intercalated MnO_2_ cathode (via electrochemically intercalating Zn^2+^ into β-MnO_2_) was used as the sole Zn source. Zn^2+^ ions were electroplated directly on the surface of the carbon nucleation layer due to the high redox potential of the Zn/Zn^2+^ couple (− 0.76 V vs. SHE) (Fig. 9a). The high electrical conductivity of the carbon nucleation layer induced homogeneous Zn nucleation and deposition. The slight lattice misfit between the carbon nanodiscs and the Zn metal and the lower energy barriers facilitated Zn nucleation and homogeneous plating/stripping. The C/Cu anode achieved a high average CE of 99.7% at 3 mAh cm^−2^ with 50 stable cycles. A high average CE at a low current density is critical in assembling reliable AF-AZMBs. The average CE of the C/Cu anode reached 97.1%, even at a low current density of 0.2 mA cm^−2^. Ensuring adequate areal capacity with a high CE is another essential factor for constructing high-energy density AF-AZMBs. The assembled anode-free battery was in the fully discharged state (Fig. 9b). When it was fully charged, Zn^2+^ ions were transferred to the anode from the pre-intercalated cathode and plated on the surface of the C/Cu anode. The battery state appeared to be similar to that with pre-deposited Zn for AZMBs (Fig. 9c). Compared with the high-capacity retention of AZMBs, the capacity of the anode-free Zn||MnO_2_ battery was maintained at 68.2% after 80 cycles owing to limited irreversible loss of Zn (Fig. 9d). Nevertheless, the energy density of the Zn||MnO_2_ battery with a Zn metal anode was only 81 Wh kg^−1^, while the energy density of the anode-free Zn||MnO_2_ battery reached 135 Wh kg^−1^ (Fig. 9e), showing the greatest advantage of anode-free structures.

Although the concept of AF-AZMBs has been proposed, the effect of protecting anodes with simple nanocarbon coatings is not ideal. How to improve the CE is the primary problem to be faced. In addition to using carbon as the protective layer, multifunctional carbon composite materials are more potent for AF-AZMBs. MXenes (e.g., Ti_3_C_2_T_*x*_) are widely used in building Zn-based anodes, which exhibit excellent mechanical properties, ample hydrophilicity, high electrical conductivity, and good ionic adsorption capacity [208]. Chen et al. prepared a Ti_3_C_2_T_*x*_/nanocellulose (derived from soybean straw) hybrid film by simple solution casting and used it as a Zn-free anode in an aqueous hybrid Zn–Li battery (Fig. 9f) [199]. The ultralow diameter of the nanocellulose provided a membrane with excellent electrolyte wettability. The nanocellulose formed pillars to prevent restacking of the Ti_3_C_2_T_*x*_. The abundant hydroxyl groups interacted strongly with Zn^2+^ ions to limit two-dimensional diffusion during the Zn deposition process. Additionally, hydrogen bonding promoted dissolution of Zn hydrate ions to reduce the Zn nucleation overpotential and promote the uniform Zn deposition. Meanwhile, the weak zincophilicity and the low lattice mismatch between the Ti_3_C_2_T_*x*_ (002) surface (2D Ti_3_C_2_T_*x*_ surface) and the Zn (002) surface induced Zn epi-plating along the horizontal [002] direction. The synergistic effect of nanocellulose and Ti_3_C_2_T_*x*_ inhibited the growth of Zn dendrites and reduced the occurrence of side reactions, which led to a higher CE for Zn plating/peeling. As shown in Fig. 9g, an anode-free flexible quasi-solid-state battery cycled at 1 A g^−1^. Only a slight capacity decay was observed with increasing bending angles, indicating that the battery was highly resistant to deformation. Moreover, Ti_3_C_2_T_*x*_/nanocellulose hybrid films were coated onto the stainless-steel foils (SS-TN80) used as the Zn-free anode in an aqueous hybrid Zn–Na battery [161]. The Na_3_V_2_(PO_4_)_3_ cathode and a “water-in-salt” (18 M NaClO_4_ + 2 M Zn(ClO_4_)_2_) aqueous electrolyte were used to construct a hybrid Zn–Na battery. The high-concentration electrolyte salts prevented freezing of the battery, which tolerated low-temperature working environments. Moreover, since the Na_3_V_2_(PO_4_)_3_ cathode did not contain Zn^2+^ ions and did not pre-intercalate Zn^2+^ ions after preparation, the Zn^2+^ ions in the electrolyte were the sole Zn source. During the first cycle, Zn^2+^/Na^+^ ions were co-intercalated and co-extracted in the Na_3_V_2_(PO_4_)_3_ cathode.

For further practical application, the influence of different environmental factors such as temperature on battery performance should be studied when improving the cycling life of high energy density AF-AZMBs. Qian et al. prepared an aluminum hydroxide fluoride (AOF) layer on the Cu foil surface as surface modification of the Cu collector anode [201]. The AOF layer exhibited strong adsorption and binding energy and accelerated the Zn desolvation and regulated the Zn^2+^ ion flux (Fig. 9h). In addition to the uniform Zn^2+^ ion concentration on the surface, the uniformly distributed current density on the AOF surface suppressed the peak effect of Zn growth, which promoted the uniform Zn deposition (Fig. 9i). Meanwhile, the low diffusion energy barrier for the AOF surface promoted 2D diffusion and growth of Zn. These advantages of the Cu@AOF electrode provided stable cycling performance even at low temperature (− 20 °C) and high average CE (99.76%) at 500 cycles. The anode-free cells were stable for 2000 cycles (Fig. 9j) and displayed a long lifespan of 400 cycles with a high average CE of 99.94% at − 20 °C (Fig. 9k). The effective protection from the Cu@AOF enabled excellent cycling of the AF-AZMBs, which showed the great potential of AF-AZMBs for use in practical applications.

Unlike the protective layer on the surface of Zn foil, the protective layer of the anode in AF-AZMBs requires close contact with the collector surface and should have a significant binding energy with the deposited Zn. This places higher demands on the protective layers of AF-AZMBs for two main reasons. First, when Zn is deposited below the protective layer, the artificial coating may break down and peel off due to the weak bonding energy of the deposited Zn. Second, the bonding energy between the protective layer and the collector should also be considered, as the protective layer will be in complete contact with the collector at the beginning of battery assembly and subsequently during the complete charge and discharge process (100% DOD). In addition, further consideration of the interface between the collector and the deposited Zn is necessary. The collector materials are weakly zincophilic but often have surface defects such as scratches and pits. Therefore, in addition to coating with the protective layer, it is possible to construct an interfacial zincophilic layer between the collector and the protective layer with a high binding energy to the collector so that the Zn is deposited uniformly and densely on the surface of the collector.

#### Anode Surface Alloying Engineering

As noted above, researchers have directly coated a protective layer on the current collector surface to inhibit side reactions and the formation of Zn dendrites while achieving a high CE and superior performance of AF-AZMBs. It is simple and convenient to operate. However, the protective layer is not bound tightly to the current collector, and there is a risk of breaking and peeling during repeated cycling. Eventually, the anode loses protection. Uniform nucleation of Zn can be realized by alloying the Zn plating/stripping interface with a zincophilic material to generate a uniform electric field on the anode.

**Fig. 10:**
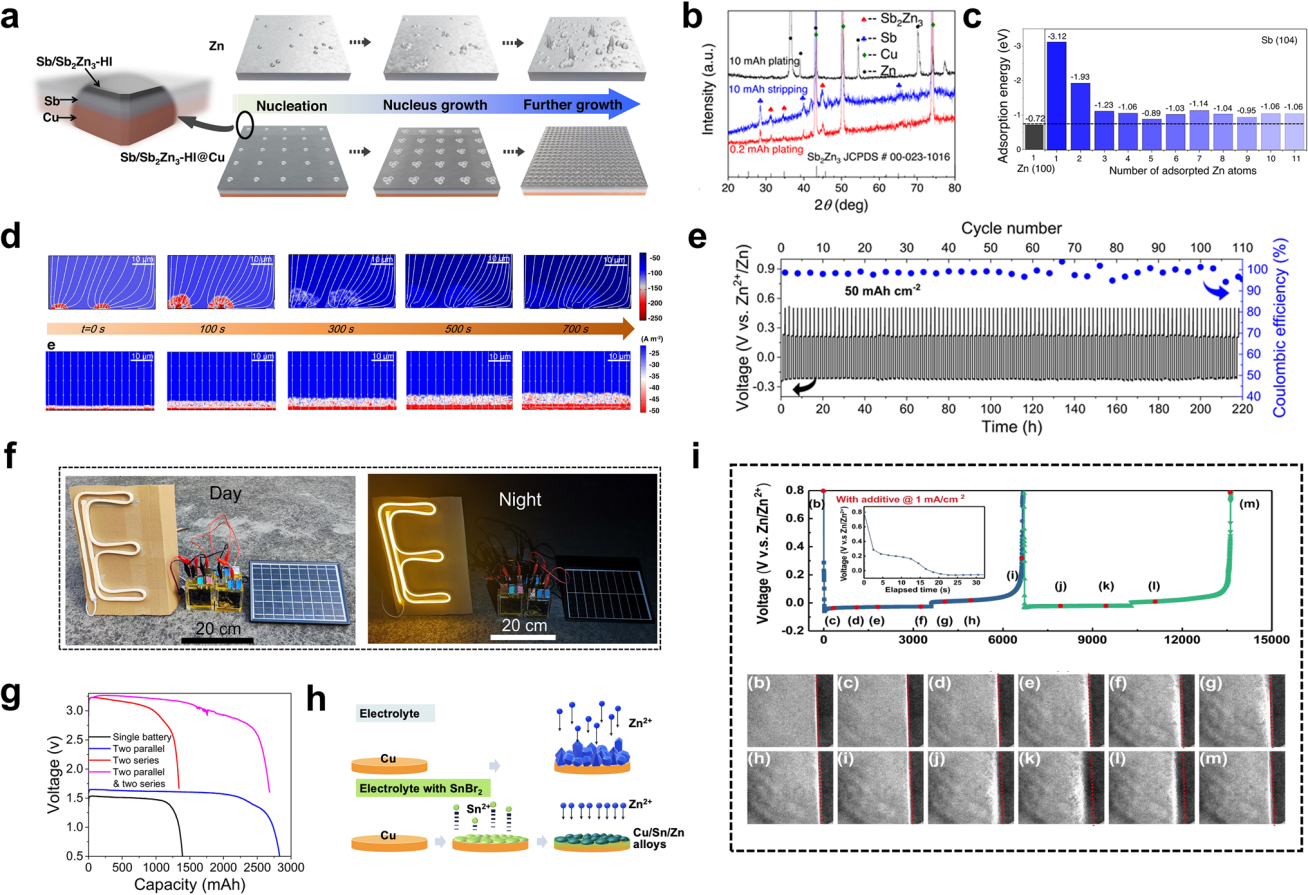
**a** Schematic illustrations of Zn electrodeposition on Zn and Sb/Sb_2_Zn_3_-HI@Cu substrates. **b** XRD patterns of the Zn electrodeposition and stripping on Sb@Cu at different capacities. **c** Adsorption energy of Zn atoms on Zn (100) and Sb (104) crystal planes. **d** Simulated current density distribution of Zn plating on Zn substrate. **e** Cycling performance of the Zn|Sb@Cu half-cell with high areal capacity and current density (50 mAh cm^−2^, 50 mA cm^−2^), and a cut-off voltage of 0.5 V versus Zn^2+^/Zn. **f** Solar powered battery energy storage system at day and night. Adapted from Ref. [203]. Copyright 2023, Open access. **g** Discharge curves of the batteries with different connections. **h** Schematic illustration of the working mechanisms of electrolyte without and with SnBr_2_. **i** TXM analysis of the first and second cycle curves of in situ Zn||Cu cell of electrolyte w/o additive and corresponding in-operando TXM images of Zn plating and stripping. Adapted from Ref. [204]. Copyright 2023, Elsevier

Chen et al. designed a robust two-dimensional heterostructural interface (Fig. 10a) [203]. Antimony (Sb) was used to form a Cu_2_Sb phase on the surface of a Cu electrode, which promoted strong bonding between the two metals and formed a robust Sb/Cu interface (Sb@Cu). When Zn was electroplated on the Sb@Cu, the Zn spontaneously alloyed with the Sb and formed an Sb/Sb-Zn heterostructural interface (Sb/Sb_2_Zn_3_-HI). XRD patterns showed that the Sb_2_Zn_3_ alloy phase formed during the initial plating stage and when the plating capacity reached a high capacity of 10 mAh cm^−2^. The characteristic peaks for the alloy phase disappeared, and those of metallic Zn were significantly enhanced (Fig. 10b). This indicated that the alloying process occurred only at the Sb@Cu surface and subsequently directed uniform Zn deposition. However, the Sb_2_Zn_3_ alloy phase remaining after Zn stripping was the complete Sb@Cu substrate, implying that the Sb/Sb_2_Zn_3_-HI formed in the initial Zn electrodeposition stage was retained in Sb@Cu and continued to regulate Zn nucleation in subsequent cycles. Initial Zn/Sb alloying enabled strong adsorption of Zn atoms by the Sb/Sb_2_Zn_3_-HI and formed a uniform electric field in the Zn coating, which resulted in uniform Zn nucleation on the Sb/Sb_2_Zn_3_-HI and consistently promoted uniform Zn deposition (Fig. 10c, d). Thus, the Zn|Sb@Cu asymmetric cell showed stable cycling over 220 h at ultrahigh areal capacity and maintained an average CE of 98.3% at 50 mA cm^−2^ (Fig. 10e). The excellent performance offered the possibility for commercial applications of AF-AZMBs. Therefore, they assembled an anode-free ZnBr_2_ pouch cell with Sb@Cu as the anode, carbon felt (CF) as the cathode, and inexpensive ZnBr_2_ and tetrapropylammonium bromide (TPABr) as the electrolyte. The reactions of the battery were as follows:

11 $${\text{Cathode:}}\;{\text{TPABr}} + 2{\text{Br}}^{-} \rightleftharpoons {\text{TPABr}}_{3} + 2e^{-}$$

12 $${\text{Anode:}}\;{\text{Zn}}^{2 + } + 2e^{-} \rightleftharpoons {\text{Zn}}$$

13 $${\text{Overall:}}\;{\text{TPABr}} + 2{\text{Br}}^{-} + {\text{Zn}}^{2 + } \rightleftharpoons {\text{TPABr}}_{3} + {\text{Zn}}$$

The cell exhibited a high energy density of ∼ 274 Wh kg^−1^. More importantly, in further attempts to develop anode-free ZnBr_2_ batteries, the capacities of the ZnBr_2_ cells were increased to 500 mAh with an alternating electrode stacking structure. A ZnBr_2_ cell was prepared with two pairs of electrodes with separate areas of 36 cm^−2^ (corresponding to a surface area of ∼ 7 mAh cm^−2^). The cell exhibited 400 stable cycles with an average CE of 98.5%. Moreover, batteries with different combinations still exhibited good electrochemical performance. Four Zn–Br_2_ cells operated at 1500 mAh were concatenated into a module exhibiting approximately 9 kWh (6 W and 1.5 mAh), which was then charged by photovoltaic panels (6 W and 9 V) during the day for approximately 2 h (Fig. 10g). The solar cell module continuously lit a 10-W LED display at night (Fig. 10f). In conclusion, this work opened a new stage and moved AF-AZMBs from the initial exploration stage toward practical application. Huang et al. utilized SnBr_2_ as additive to form an in situ Cu/Sn/Zn alloy anode on a Cu current collector and assembled an anode-free aqueous hybrid Cu||LFP full cell (Fig. 10h) [204]. They optimized the electrolyte and cathode capacity (E/C) ratio to obtain better electrochemical performance for AF-AZMBs. Moreover, they utilized in-operando transmission X-ray microscopy (TXM) measurements to determine the mechanism for in situ Zn and Sn plating/stripping at the Cu collector current. The temporal points selected during the events were marked with their corresponding TXM images (Fig. 10i). The electrolyte containing the SnBr_2_ additive formed an in situ Sn coating at the anode surface and a well-nucleated alloy with Cu to guide uniform and smooth Zn deposition. The Cu/Sn/Zn alloy promoted nucleation and dense and uniform Zn deposition. The reactions of the assembled anode-free aqueous hybrid Cu||LFP full cell were as follows:

14 $${\text{Cathode:}}\;{\text{LiFePO}}_{4} \rightleftharpoons {\text{Li}}^{ + } \left( {{\text{aq}}.} \right) + {\text{FePO}}_{4} + e^{-}$$

15 $${\text{Anode:}}\;{\text{Zn}}^{2 + } \left( {{\text{aq}}.} \right) + 2e^{ - } \rightleftharpoons {\text{Zn}}\left( {\text{s}} \right)$$

This meant that Zn only affected the anode, and intercalation/removal of the Li^+^ contributed to the cathode capacity.

#### Anode 3D Structural Design

**Fig. 11:**
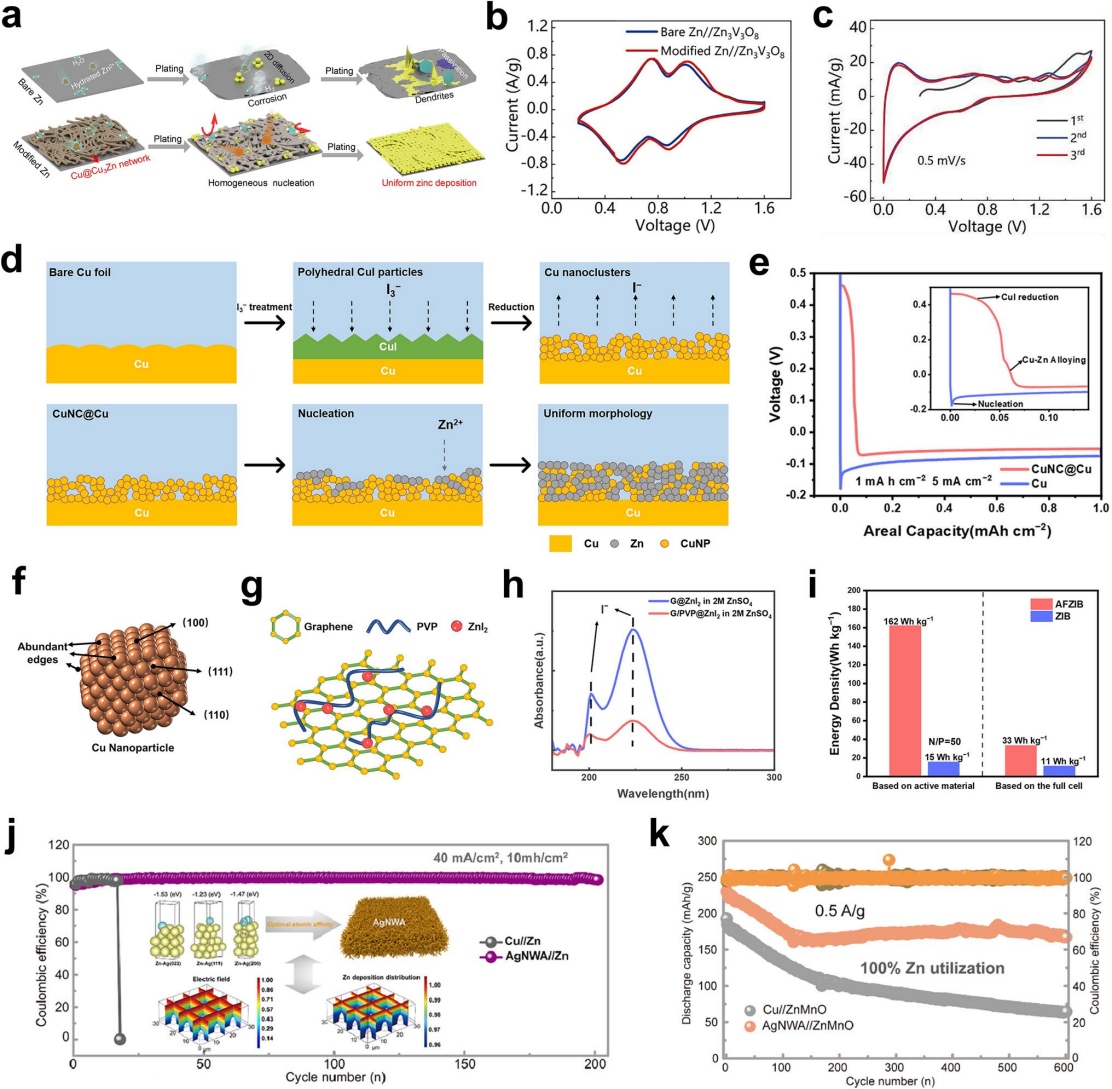
**a** Schematic illustrations of Zn deposition behaviors on the surface of bare Zn foil and the Cu@Cu_3_Zn network-modified Zn foil. **b, c** CV curves of the AF-AZMBs. Adapted from Ref. [205]. Copyright 2023, Elsevier. **d** Schematic illustrations of the surface morphological changes of the Cu foil during in situ reconstitution and the Zn deposition behavior on CuNC@Cu. **e** First-cycle galvanostatic deposition curve on CuNC@Cu and Cu electrodes at 5 mA cm^−2^, the different deposition process of two electrodes in the inset. **f** Schematic illustration of (100), (110), (111) planes and abundant facet edges of Cu nanoparticles. **g** Advantages of ZnI_2_ cathode with G/PVP host. **h** UV–Vis absorption spectra of the ZnSO_4_ electrolyte after immersing the full-charged G/PVP@ZnI_2_ or G@ZnI_2_ cathode for 24 h. **i** Comparison of gravimetrical energy density of AFZIB and ZIB based on the mass of active material and the mass of full cell. Adapted from Ref. [196]. Copyright 2022, Open access. **j** CEs of Cu||Zn and AgNWA||Zn cells at 40 mA cm^−2^ and 10 mAh cm^−2^. **k** Cycling curves of AgNWA||ZnMnO and Cu||ZnMnO anode-free full batteries with 100% Zn utilization. Adapted from Ref. [197]. Copyright 2022, Elsevier

Compared with the coating protection layer of a 2D planar structure, the 3D structure anodes can adapt more readily to the drastic volume changes caused by Zn metal deposition/stripping, thus improving the cycling stability of Zn anodes. The zincophilic sites in the 3D structure control the nucleation energy and provide uniform deposition of Zn [209]. In addition, the 3D structure homogenizes the local electric field on the surface [207]. The large contact interface between the electrode and the electrolyte balances the Zn^2+^ flux along the anode surface, which provides sufficient charge centers and nucleation sites. Dong et al. designed a 3D Cu/Zn alloy network (Cu@Cu_3_Zn) for Zn deposition (Fig. 11a) [205]. They found that the electrochemical behavior of AF-AZMBs differed significantly from those of conventional AZMBs in CV experiments (Fig. 11b, c). It is certain that the reduced Zn contents in different batteries, i.e., AF-AZMBs with limited Zn, significantly alter the electrochemical behavior during Zn deposition/stripping. Therefore, it is critical to improve the area capacity of the anode in AF-AZMBs and compare the electrochemical characteristics. Lu et al. used a Cu foil as the deposition substrate and added a trace amount of I_3_^−^ (10 mM) to the aqueous ZnSO_4_ electrolyte [196]. The strongly oxidizing I_3_^−^ reacted quickly with the Cu surface to form copper iodide (CuI). In the Cu/Zn half-cell, CuI was electrochemically reduced in situ and reconstructed into porous Cu nanoclusters (CuNCs) from the outside (Fig. 11d). For the CuNC@Cu electrode, Cu and Zn alloying and dealloying were observed during the formation of copper nanoparticles (Fig. 11e). In addition to the typical (100), (110), and (111) planes, the Cu nanostructures with high specific surface areas had more exposed edges than the Cu foil, and the abundant zincophilic sites on these edges lowered the Zn nucleation barrier (Fig. 11f). In addition, due to the number of exposed zincophilic sites in CuNCs and the ample space for Zn deposition, the Zn showed a sheet morphology and was horizontally distributed on the surface of the CuNCs. Moreover, numerous Cu nanoparticles were dispersed between the Zn sheets, thus forming a horizontally stacked Zn/Cu composite structure. Subsequently, an anode-free G/PVP@ZnI_2_||CuNC battery with a ZnI_2_ cathode was assembled (Fig. 11g). After the immersion pre-cycles, the G/PVP@ZnI_2_ cathode was placed in a 2 M ZnSO_4_ electrolyte for 24 h, and UV spectroscopy showed that immersion of the G/PVP@ZnI_2_ cathode in the electrolyte generated a significantly weaker iodine absorption peak, indicating that the G/PVP effectively inhibited the shuttle effect (Fig. 11h). The assembled anode-free Zn||I_2_ batteries (AFZIBs) had gravitational energy density of 162 Wh kg^−1^ (based on the total mass of the active material), which was much larger than the 15 Wh kg^−1^ value of the Zn||I_2_ batteries (ZIBs) with the Zn foil anode (Fig. 11i).

In addition to the number of zincophilic sites, it is also important to establish a uniform electric field on the 3D anode surface. With increasing current density or deposition capacity, nonuniform Zn deposition was increasingly evident due to the limited influence of these modifications. Moreover, base metals that provide zincophilic sites should also be selected. In short, the higher binding energy between an adsorbed Zn atom and the surface site contributes to the overpotential of the Zn metal deposition process. A strong binding energy between the adsorbed Zn atoms and the substrate enhances the resistance of the Zn metal stripping process on the substrate, leading to inhomogeneous Zn deposition/stripping behavior. In addition, as mentioned earlier, one of the essential indicators for AF-AZMBs is high and stable CE with an ultrahigh current density and surface capacity during long cycling. However, in the case of a large current and large surface capacity, the utility of the protective layer with a 2D structure is very limited. With increasing current density or deposition capacity, the influence of Zn on these protective layers continues to weaken after continuous electroplating on the anode surface, resulting in uneven Zn deposition. Huang et al. constructed a 3D light silver nanowire aerogel (AgNWA) via vertical self-assembly (Fig. 11j) [197]. The zincophilic silver metal substrate showed the highest Zn adsorption energy. The silver nanowires in the AgNWAs enabled rapid electron conduction at the interface. The cross-linked network provided a uniformly distributed electric field for evenly reversible Zn deposition/stripping. The Zn deposition simulation showed highly reversible and smooth Zn metal deposition on the 3D AgNWAs. In addition, the 3D AgNWAs with a porous structure maintained close contact with the electrolyte and exhibited good hydrophilicity while limiting the sharp volume changes of Zn metal deposition/stripping. This promoted uniform Zn metal deposition/stripping during the cycling process. The AgNWA anodes achieve dendrite-free Zn deposition with ultrahigh current density and capacity (40 mA cm^−2^, 10 mAh cm^−2^) and high CE (99.8%). The AgNWAs were directly coupled with a pre-galvanized MnO_2_ cathode (ZnMO), which formed a complete AF-AZMB. The anode-free AgNWA||ZnMO battery provided an initial capacity of 230 mAh g^−1^ and a high capacity retention rate of 73% at 0.5 A g^−1^ after 600 cycles with a 100% Zn utilization (Fig. 11k).

When the limited Zn in the cathode is deposited to the anode, it is important for AF-AZMBs to minimize the Zn loss and promote the uniform Zn deposition. Therefore, the collector of AF-AZMBs should possess good electrical conductivity and zincophilicity and can inhibit the dendrite growth and side reactions as much as possible. Strategies such as surface coating engineering, surface alloying engineering, and 3D structure design can reduce the energy barrier of Zn deposition, promote the uniform Zn nucleation, decrease the Zn loss during cycling, and improve the CE. In addition, in order to further increase the energy density of AF-AZMBs, it is necessary to further reduce the proportion of current collector in the full cell.

### Electrolyte Engineering

The electrolyte directly contributes to anode/electrolyte interface problems, such as dendrite growth, HER, and the formation of byproducts [210, 211]. For AF-AZMBs, the importance of the electrolyte is more prominent. Zn^2+^ ions in the electrolyte can be used as the Zn source of AF-AZMBs, and the loss of electrolyte due to anode/electrolyte interface issues is lethal for AF-AZMBs. This leads to decreased cycle lifespan and capacity for batteries. Electrolyte optimization is a promising way to regulate the anode/electrolyte interface. In addition, reducing the amount of free H_2_O in the electrolyte is an effective and feasible strategy for mitigating side reactions and corrosion [212]. Electrolyte additives can change the conductivity of the electrode and optimize the current distribution, thereby suppressing the growth of dendrites [213, 214]. Electrolyte additives mainly play two roles, one is to form effective protective layers at the anode surface, and the other is to suppress HER by changing the solvation structure of the electrolyte.

#### Anode/Electrolyte Interface Design

**Fig. 12:**
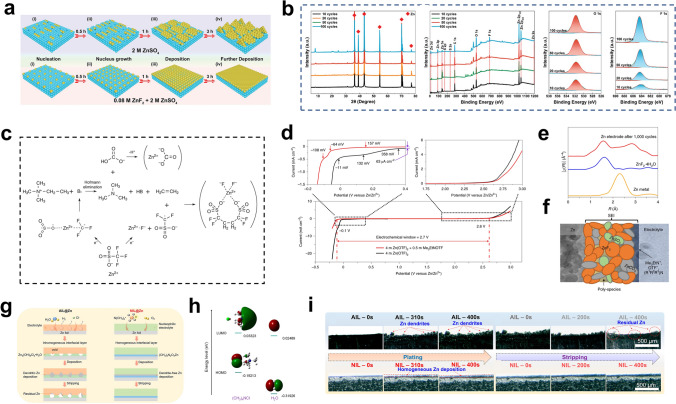
**a** Schematic illustration for depositing Zn without/with ZnF_2_ electrolyte addictive. **b** XRD patterns and spectra of Zn anodes at different cycles with ZnF_2_ electrolyte addictive. Adapted from Ref. [210]. Copyright 2021, John Wiley and Sons. **c** Mechanism of synergistic reactions to deposit predominantly fluoride and carbonate-based SEI. **d** Electrochemical stability windows of 4 M Zn(OTF)_2_ and 4 M Zn(OTF)_2_ + 0.5 M Me_3_EtNOTF electrolytes. **e** X-ray absorption spectroscopy fine structure data of ZnF_2_·4H_2_O and Zn electrode recovered from 4 M Zn(OTF)_2_ + 0.5 M Me_3_EtNOTF electrolyte after 1000 cycles. **f** Schematic illustration of Zn^2+^-conducting SEI. Adapted from Ref. [215]. Copyright 2021, Springer Nature. **g** Schematic illustration of the formation of interfacial layers on Zn foil in different electrolytes. **h** LUMO and HOMO isosurfaces of (CH_3_)_4_NCl and H_2_O molecules. **i** In situ optical microscopy images of the plating and stripping processes of Zn with AIL and NIL. Adapted from Ref. [216]. Copyright 2022, American Chemical Society

Additives can regulate the anode/electrolyte interfaces to manage Zn^2+^ ion mobility and Zn plating/stripping. Feng et al. added zinc fluoride (ZnF_2_) to the electrolyte to form stable F-rich interface on the anode surface (Fig. 12a) [210]. The anode was combined with a LiMn_2_O_4_ (LMO) cathode in an anode-free cell. This F-rich interface effectively reduced the nucleation overpotential and plateau overpotential for Zn deposition, thus regulating the distribution of Zn^2+^ ions and inducing uniform Zn deposition. As seen from the XRD pattern obtained for the anode after cycling, the peaks of Zn_4_SO_4_(OH)_6_H_2_O were weaker and changed little during cycling. Additionally, the XPS patterns of the anode after different numbers of cycles showed that the intensity of the *F* peak became stronger, indicating that during the cycling process, a F-rich layer was constantly formed on the anode surface, which effectively inhibited side reactions (Fig. 12b). Therefore, a high CE (99.14% after 100 cycles) was observed even with a high current density (40 mA cm^−2^) and areal capacity (3.0 mAh cm^−2^).

In addition to forming a single fluorine-rich protective interface on the anode surface, the multielement synergistic effect of the electrolyte additives in forming the SEI was also studied. One way to inhibit dendrite growth and side reactions on the anode surface is to build a Zn^2+^-conductive SEI on the anode/electrolyte interface. Wang et al. added trimethylethylammonium trifluoromethanesulfonate (Me_3_EtNOTF) to the dilute acid electrolyte (Zn(OTF)_2_) [215]. The additive formed a fluorinated and hydrophobic SEI on the collector surface. The composite SEI was composed of ZnF_2_, ZnCO_3_, ZnSO_3_, and polyanions (Fig. 12e, f). DFT calculations revealed the formation process of the SEI film. The Me_3_EtNOTF defluorination reaction generated (SO_3_)F_2_C and reacted with ethylene in a high-strength polymerization reaction. The reaction shifted radicals to the H_2_C. This was stabilized by adsorbing Zn at the interface until the second reduction product was added. In addition, the downstream reaction produced by decomposition of the alkylammonium reagent led to the formation of ZnCO_3_. The amines easily reacted with CO_2_ or H_*x*_CO_3_ (*x* = 1, 2) in the aqueous environment to form CO_3_^2−^ (Fig. 12c). The fluorinated and hydrophobic interface formed in situ prevented the HER and allowed the migration of Zn^2+^ ions. The interface enriched in ZnF_2_ promoted the diffusion of Zn^2+^ ions while protecting the Zn surface and preventing side reactions. In addition, the ZnF_2_/Zn interface inhibited dendrite growth by promoting 2D Zn^2+^ ion migration and deposition. The addition of Me_3_EtNOTF provided a wider stability window of approximately 2.7 V, and the limits of the cathode (− 0.1 V vs. Zn/Zn^2+^) and the anode (2.6 V vs. Zn/Zn^2+^) were expanded (Fig. 12d). Specifically, Me_3_EtNOTF extended the cathode limit, inhibited the precipitation of oxygen, and increased the initial oxidation potential from 2.55 to 2.6 V. Additionally, the initial potential for water reduction was reduced from 358 to 157 mV (relative to Zn/Zn^2+^) and then slightly reduced at − 64 mV. Zn plating occurred at − 108 mV. The HER current at the cathode side was eliminated. The presence of Me_3_EtNOTF promoted the formation of the SEI. After 100 cycles, the CE was increased to 99.9%, and the average value for 1000 cycles was 99.8%. To verify the role of ZnF_2_ in the SEI, a pure ZnF_2_ SEI was designed with the bis(fluorosulfonyl)imide anion (FSI^−^) as the fluorine source. This was tested in a Zn||MnO_2_ battery with limited Zn. The battery delivered a high energy density of 350 Wh kg^−1^ and capacity retention rate of 88.5% after 1000 cycles. Additionally, the electrolyte formed by the SEI also allowed the Ti||Zn_*x*_VOPO_4_ anode-free pouch cell to operate reversibly for 100 cycles at 100% DOD.

In addition to the SEI, nucleophilic reagents have been used to construct an interface protection layer in situ. The key to forming this interfacial layer is to screen nucleophilic reagents and find one with appropriate electron donor properties. Hu et al. used tetramethylammonium chloride (TMACl) as a nucleophilic agent containing N to construct a uniform nucleophilic interface layer (NIL) from Zn acetate acetamide [216]. The NIL was uniform and dense, and it conducted Zn^2+^ ions to provide dendrite-free Zn deposition (Fig. 12g). In an aqueous electrolyte comprising ZnSO_4_-LiCl, an uneven layer of Zn(OH)_2_ was formed on the anode surface due to occurrence of HER. After combination of the Cl^−^ anion and Zn(OH)_2_, the inhomogeneous alkaline Zn_5_(OH)_8_Cl_2_·H_2_O interfacial layer (AIL) was finally formed on the anode surface. Due to the low resistance, Zn^2+^ ions were preferably deposited around the thinner area of the interface layer, and the dendritic morphology of the deposited Zn was finally observed. In the nucleophilic electrolyte containing ZnSO_4_–LiCl–TMACl, Zn^2+^ ions promoted the reaction between the N(CH_3_)_4_^+^ ions and O_2_, and (CH_3_)_4_NCl had a higher HOMO (highest occupied molecular orbital) energy than H_2_O (− 0.19213 vs. − 0.31926 eV). Therefore, (CH_3_)_4_NCl provided electrons more easily than H_2_O (Fig. 12h). This caused the N(CH_3_)_4_^+^ to react preferentially with the nucleophilic electrolyte and form a Zn acetate acetamide protective layer. The uniform NIL provided more nucleation sites for Zn^2+^ ion deposition, thus promoting the uniform Zn electrodeposition. In in situ SEM studies (Fig. 12i), unlike the dendritic Zn surface observed on the cycling Zn anodes with AIL, the electroplated Zn on the NIL showed excellent uniformity. A NIL@Ti anode was prepared in the anode-free Zn–Cl_2_ battery. The electrode underwent 200 stable cycles at 20 mA cm^−2^.

#### Electrolyte Structure Design

**Fig. 13:**
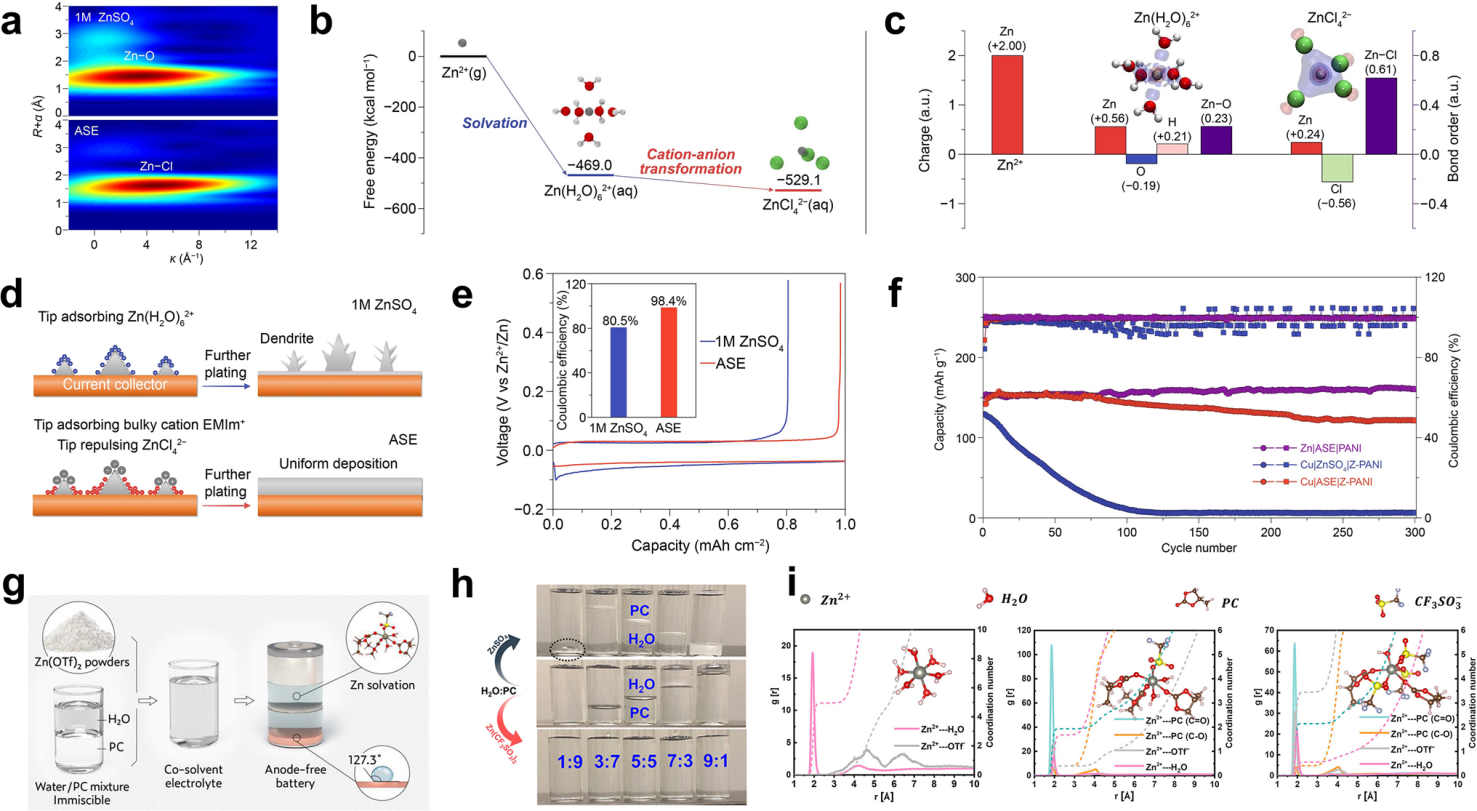
**a** EXAFS spectra by the wavelet transform. **b** Free energy change in the Zn^2+^ solvation and cation–anion transformation processes. **c** The atomic charge, bond order, and differential charge density analysis of Zn(H_2_O)_6_^2+^ and ZnCl_4_^2−^. **d** Schematic illustration of Zn plating pattern in 1 M ZnSO_4_ electrolyte and the anion-type water-free Zn^2+^ solvation structure electrolyte (ASE). **e** Voltage profiles and CEs of Zn plating and stripping processes in the first cycle. **f** Cycling performance of the anode-free cell and the Zn|ASE|PANI cell. Adapted from Ref. [217]. Copyright 2021, John Wiley and Sons. **g** Schematic diagrams of the preparation of the co-solvent electrolyte and the corresponding Zn solvation structure and the hydrophobic interphase in the anode-free battery. **h** Digital images of the water/PC mixture with the 1 M ZnSO_4_ and Zn(CF_3_SO_3_)_2_. **i** The solvation structures for 1 M Zn(OTf)_2_ in water, 50% PC-sat., and 90% PC-sat. within a 0.3 nm scale. Adapted from Ref. [218]. Copyright 2022, American Chemical Society

The CE for Zn plating/stripping is the most critical factor for stable AF-AZMBs, and it is inevitably limited by water-induced side reactions and dendrite growth. The Zn^2+^ ion solvation structure Zn(H_2_O)_6_^2+^ controls the HER on the Zn metal anode. A proper electrolyte structure replaces the original Zn(H_2_O)_6_^2+^ with a new water-free solvation structure. Chen et al. designed an anionic water-free electrolyte for Zn^2+^ ion solvation (ASE) as ZnCl_4_^2−^ by adding a chloride salt with a bulky cation (1-ethyl-3-methylimidazolium chloride) to a ZnSO_4_ aqueous electrolyte [217]. The Fourier transformation of extended X-ray absorption fine structure (EXAFS) spectra by the wavelet transform clearly present the difference in the Zn^2+^ ion coordination states (Fig. 13a). DFT calculations revealed the coordination mechanism of Cl^−^ ions (Fig. 13b). Zn^2+^ ions were strongly coordinated by Cl^−^ anions. The coordination of Cl^−^ ions released the waters bound to Zn^2+^ ions and reconstructed the electrolyte structure. The Zn(H_2_O)_6_^2+^ solvation structure was replaced by ZnCl_4_^2−^. The energy of ZnCl_4_^2−^ was lower than that of Zn(H_2_O)_6_^2+^. The transformation was a spontaneous process, and 60.1 kcal mol^−1^ was released.

16 $${\text{Zn}}\left( {{\text{H}}_{2} {\text{O}}} \right)_{6}^{2 + } \left( {{\text{aq}}} \right) + 4{\text{Cl}}^{-} \left( {{\text{aq}}} \right) \to {\text{ZnCl}}_{4}^{{2{-}}} \left( {{\text{aq}}} \right) + 6{\text{H}}_{2} {\text{O}}\left( {{\text{aq}}} \right) + 60.1\,{\text{kcal}}$$

In addition, compared with the Zn centers in ZnCl_4_^2−^ and Zn(H_2_O)_6_^2+^, the Zn atomic charge in ZnCl_4_^2−^ (+ 0.24) was lower than that in Zn(H_2_O)_6_^2+^ (+ 0.56), suggesting that Cl^−^ was a more potent electron donor for Zn^2+^ than H_2_O. The Zn–Cl bond order (0.61) was much higher than that of the Zn–O_W_ bond (0.23), and the electron density around the Zn in ZnCl_4_^2−^ was higher than that around the Zn in Zn(H_2_O)_6_^2+^, demonstrating a more robust interaction between the Zn^2+^ and Cl^−^ (Fig. 13c). During the Zn plating process, adsorption of the bulky cation EMIm^+^ inhibited Zn plating on the tips, and ZnCl_4_^2−^ was repelled by the electrostatic shield on the Zn tips. The ZnCl_4_^2−^ anion was only deposited via 2D diffusion in a weak electric field (Fig. 13d). Therefore, growth of dendrites at the Zn tips was inhibited, and the electrolyte showed uniform Zn deposition with an average Zn plating/stripping CE of ≈ 99.9% (Fig. 13e). The anode-free battery with a Cu foil anode, a Zn polyaniline (Z-PANI) cathode, and the ASE electrolyte exhibited a high capacity of 154.4 mAh g^−1^ with a capacity retention rate of 78.8% after 300 cycles (Fig. 13f).

Alshareef et al. proposed a salting-in-effect-induced hybrid electrolyte by adding Zn triflate (Zn(OTf)_2_) into propylene carbonate (PC)/water mixtures (Fig. 13g) [218]. PC is immiscible with water and most Zn salts. An apparent stratified phase was observed when the PC content was higher than 16.7 vol%. After adding 1 M ZnSO_4_, the ZnSO_4_ attracted the water dissolved in the PC due to the salting-out effect, resulting in enhanced liquid phase separation. The salting-in effect occurred when 1 M Zn(OTf)_2_ was added to the mixed solvent, which made the initially miscible mixture completely miscible (Fig. 13h). The radial distribution function (RDF) plots showed that PC and OTf^−^ replaced the water and participated in the formation of Zn^2+^-solvated shells (Fig. 13i). Triflate anions contain the strongly hydrophobic –CF_3_ group and the hydrophilic –SO_3_^−^ group. Anions coordinated by the PC (or water) were dispersed in the solvent as [PC–OTf^−^–H_2_O]amphipathic complexes present in the Zn^2+^ solvation sheath in the presence of PC. After adding the PC, the Zn(H_2_O)_6_^2+^ was replaced by OTf^−^ and PC. In addition, different PC concentrations resulted in different structures of the Zn^2+^-solvated sheaths. When the PC content was 50 vol%, only two water molecules were present in the first solvation layer of each Zn^2+^, and PC and OTf^−^ were predominant. When the PC content was increased to 90 vol%, the solvating water molecules were replaced by PC or OTf^−^. The substitution of solvent water reduced the water activity in the hybrid electrolyte and inhibited the occurrence of side reactions. Moreover, the unique solvation structure resulted in the reduction of anions, thus forming a hydrophobic SEI that achieved uniform galvanizing and prevented side reactions. The presence of the hybrid electrolyte in the Zn^2+^ solvent shell and the formation of the hydrophobic electrode/electrolyte SEI inhibited side reactions and excess dendrite growth, which gave the Zn^2+^ a high CE for electroplating/stripping and was crucial for AF-AZMBs.

In situ SEI can regulate the deposition behavior of Zn^2+^ ions, inhibit side reactions and Zn dendrites, and reduce the Zn loss during cycling. The change of electrolyte structure is conducive to inhibiting HER and regulating Zn deposition kinetics. However, the formation of SEI inevitably loses some Zn^2+^ ions, which should be avoided for AF-AZMBs. Moreover, the complex electrode surface renders it more difficult to investigate the interfacial reaction mechanism. To further expand the application of AF-AZMBs, it is necessary to develop gel electrolytes and solid electrolytes for AF-AZMBs. The synergistic modification strategy of electrolyte and collector is a potential direction to improve the performance of AF-AZMBs.

Although AF-AZMBs offer advantages such as high energy densities and low initial energy states, there are stricter requirements for Zn electroplating/stripping efficiency at the anode. The main problem with the anodes of AF-AZMBs is the “dead Zn” formed by excess dendrite growth and the by-products produced by the side reactions, which result in low CEs for electroplating/stripping and limit the cycling life of the battery [54, 197]. In addition, preventing the decomposition of the electrolyte during the cycling process is the key to improving the Zn utilization [218]. Therefore, we will summarize the challenges associated with the anode of AF-AZMBs and propose solutions to improve the CEs and cycling performance.

To overcome the problems of AF-AZMBs, we first need to classify them. Based on the different sources of Zn, they can be divided into electrolyte Zn source anode-free Zn metal batteries (ES-AFZMBs) and cathode pre-embedded Zn (Zn source form cathode) anode-free Zn mental batteries (CS-AFZMBs). The ES-AFZMBs are more convenient than the CS-AFZMBs, which must be pre-embedded with Zn in advance. However, during the first discharge cycle, side reactions such as water electrolysis are more likely to occur on the cathode side [55]. Based on different reactions on the cathode, the AF-AZMBs can be divided into traditional Zn^2+^ ion-embedded/detached and new AF-AZMBs with different cathode electrochemical reactions, such as Zn–Li [199, 204, 214], Zn–Na [200], Zn–Br_2_ [203, 206], Zn–Cl_2_ [216] and Zn–I_2_ [196, 213]. Most of the Zn sources in the new battery system come from the Zn-rich electrolyte, so it belongs to ES-AFZMB system. This overcomes the lack of a suitable Zn-rich cathode. Nevertheless, the electrochemical processes of cathode in different systems will also bring new problems, such as the shuttle effect of iodine and the diffusion of liquid bromine. These problems make single modification strategy of the anode or electrolyte insufficient. Therefore, in constructing AF-AZMBs, the cathode/electrolyte interface reaction must be regulated with multiple or multifunctional modification strategies. At present, the lack of Zn-rich cathodes may be the main reason for constructing various new systems in AF-AZMBs [54]. For CS-AFZMBs, similar to the “bucket effect”, the low Zn capacity of the cathode seriously limits the energy density of AF-AZMB. The concentrations and cost of Zn^2+^ ions in aqueous electrolytes also limit the construction of AF-AZMB with high energy densities.

## Conclusions and Perspectives

Aqueous Zn metal batteries have received considerable attention due to their high energy densities, intrinsic safety, and low costs. However, compared with the successful industrialization and market dominance of lithium-ion batteries, the commercialization of AZMBs is still a long way off. One of the main reasons is the need for excess Zn in the Zn metal anode, which leads to the ultralow Zn utilization, resulting in large gaps between the actual and theoretical energy densities of AZMBs. Therefore, limiting the use of excess Zn while effectively suppressing dendrite formation and side reactions has become an urgent problem. In this review, we have discussed in depth how to design AZMBs with high Zn utilization along the main line of reducing the amount of excess Zn, from utilizing thinner Zn foils to constructing anode-free structures with theoretical Zn utilization of 100%:

1. Thinner Zn foils increase the Zn utilization. However, the Zn lost during cycling cannot be replenished promptly, and the defects on the thin Zn foil surface are magnified and expose it to the risks of shattering and rapid cell failure. An effective protection strategy to minimize Zn loss during cycling becomes more critical in such a situation. Zn losses are mainly due to the formation of "dead Zn" from the overgrowth of dendrites produced by a nonuniform electric field on the anode surface and the byproducts of HER, corrosion, and passivation processes. Strategies such as artificial interface protection, electrolyte additives, and separator design protect the thin Zn foil anode at high Zn utilization and promote uniform and dense Zn deposition. Depending on the material, artificial interface protection layers can be classified as inorganic salts, metals and their alloys, carbon materials, and polymers. Electrolyte additives can be classified by the mode of action as solvent–water structural modification, EDL modulation, in situ SEI formation, and hydrogel electrolytes. Separator modifications can be classified into glass fiber surface modifications and new material separator construction. These strategies effectively protect the thin Zn foil anodes in various ways, resulting in high Zn utilization with stable cycling performance.
2. Pre-depositing active Zn on a collector to form the anode is an effective strategy to improve the Zn utilization of the Zn anode. Constructing a 3D structured collector is the primary protection strategy for pre-deposited Zn. They can be classified as carbon materials (e.g., CNTs and 3D-printed graphene), metal materials, and 2D MXene and MOF derivatives. These 3D structured materials reduce the energy of Zn formation through internal uniform distribution of the zincophilic sites. The uniformly distributed pore structure and good electrical conductivity enable uniform distribution of the electric field and Zn ion flux, which promotes uniform Zn deposition.
3. AF-AZMBs have been proposed to maximize the Zn utilization. The Zn in an AF-AZMB is pre-distributed in the electrolyte or cathode, at which point there is no Zn on the anode. Theoretically, for AF-AZMBs with N/P = 1, the Zn^2+^ in the electrolyte or cathode will be deposited on the anode collector during the first charge and utilized in subsequent cycles when the Zn utilization reaches 100%. However, in practice, since the Zn content is minimal, even a minor loss of Zn can significantly reduce the cycling life of the battery. As a result, AF-AZMBs often require high CEs. This means that side reactions and the growth of Zn dendrites during cycling must be suppressed as much as possible. Therefore, higher demands are placed on the anode protection strategy. For collector modification, the main strategies are surface coating, alloying, and 3D structural design. For electrolyte additives, protection strategies have been proposed for both electrode/electrolyte interface design and electrolyte structural design. Although research on AF-AZMBs is still in its initial stages, these strategies provide new directions for the development of AF-AZMBs.

Although significant progress has been made in constructing Zn metal anodes with high utilization, some problems still need to be solved. Therefore, in order to further reasonably reduce the amount of excess Zn and designing AZMBs with high Zn utilization, our viewpoints and recommendations are shown below:

- While achieving a high Zn utilization and good performance at high DOD (in symmetrical cells) with an overly thick Zn foil is possible, this will result in the need to increase the capacity or load of the matched cathode when assembling a full cell if a low N/P is desired. An overly thick cathode will result in slow ion migration kinetics and high impedance, reducing cathode utilization. A full cell assembled with a thicker cathode will result in excess use of the Zn anode due to the limited cathode utilization, even if a low N/P is achieved. Further optimization of the cathode is required to achieve a high energy density with thick Zn foils. Therefore, the load of the cathode should also be increased to unify the anode and cathode while improving the Zn utilization by reducing the thickness of the Zn foil or controlling the Zn capacity by pre-deposition.
- The amount and form of the Zn in the anode should be consistent during material characterization and battery performance tests. Some researchers have investigated the electrode/electrolyte interfacial properties and Zn deposition behavior when using a Zn foil as the anode and achieved a high Zn utilization. When assembling full cells, a certain amount of Zn in the collector (such as carbon cloth and Cu foil) is pre-deposited to serve as the anode and achieve a high energy density. This leads to further decreases in the N/P. Nevertheless, the change in Zn deposition and the environment at the anode/electrolyte interface must be investigated due to the shift from Zn foils to pre-deposited Zn anodes.
- The purpose of reducing the excess Zn in the anode is to reduce the N/P for the full cell, increase the energy density, and promote industrial application of AZMBs. In addition to reducing the excess Zn, the inactive collector and separator must be optimized to improve the energy density, such as by using lighter materials or adjusting the 3D structure of the collector to reduce its mass and volume or reducing the thickness of the diaphragm while maintaining its mechanical properties. Additionally, industrialization of AZMBs also requires controlling the manufacturing cost. For the anode, preparation with some protection strategies is very complicated, which undoubtedly increases the manufacturing cost of the battery. Therefore, further optimization of the material preparation process is needed.
- A synergistic combination of multiple protection strategies could be applied. Given the relationships among dendrite overgrowth and the various side reactions occurring at the Zn metal anode, more reliance on diversified protection strategies is needed. In addition, the anode/electrolyte interface becomes more unstable as the Zn utilization at the anode increases. The synergistic effect of multiple protection strategies can simultaneously control the effects of multiple factors on the Zn deposition process. For example, establishing a multigradient anode with hydrophilic and zincophilic gradients could promote uniform Zn deposition while isolating water from the metal anode surface. In addition, in situ growth of a protective hydrogel electrolyte layer with a powerful binding force on the surface of the metallic Zn anode would eliminate the need for a septum while protecting the Zn anode.
- For AF-AZMBs, we have three recommendations for assessing the performance of anode-free cells: (1) First, the *E*/*C* ratio must be introduced [where *E* is the electrolyte capacity (mAh), and *C* is the areal capacity of the positive electrode (mAh)]. While the electrolyte is the only Zn source, the *E*/*C* cannot be kept as close to 1 as it is with N/P because Zn^2+^ ions are not deposited entirely on the surface of the anode to contribute 100% of the capacity. At this point, calculation of the Zn utilization for an AF-AZMB should be based on the capacity of the Zn deposited on the negative electrode during the first charge. (2) The next step is to tighten the test conditions for anode-free batteries with high area capacities, which are the critical indicators needed to move anode-free batteries from the laboratory to practical application. In addition, more attention should be given to changes in the electrochemical behavior of the anode surface arising from Zn depletion at high current densities and high area capacities, which may improve the CE for electroplating. (3) The CE for the first plating/stripping cycles should be improved. The magnitude of the CE directly affects the Zn utilization of the anode-free cell. Most anode-free cells lose a significant amount of Zn during the first plating/stripping cycle, significantly reducing the energy density. Second, the average CE should also be considered. In addition, double ion cells, rocker cells, and electrode-less cells, which also use limited amounts of Zn, are gaining interest for increasing the energy density of the cell.
- Improving the Zn utilization is an inevitable requirement for the further development of AZMBs. What is the optimal Zn utilization for AZMBs? It depends on the usage scenario and market demand of AZMBs. In other words, optimal Zn utilization is required to meet the economic cost and energy density. As a candidate for the next generation of large-scale energy storage, AZMB systems have relatively low energy density requirements for an individual cell, so the Zn utilization does not need to be increased to 100%. For other applications such as wearable electronics, AZMBs with high Zn utilization, especially AF-AZMBs with 100% Zn utilization, just meet the requirements of high energy density.

Although there are still many challenges in developing AZMBs with high Zn utilization, outstanding research results are constantly emerging. It is believed that with the continuous innovation of anode-free structures and the deepening of mechanism research, designing and constructing commercialized AZMBs with high Zn utilization of 100% will ultimately be achieved.
